# Loss-of-function mutations in the fruit softening gene *POLYGALACTURONASE1* doubled fruit firmness in strawberry

**DOI:** 10.1093/hr/uhae315

**Published:** 2024-11-19

**Authors:** Nicolás P Jiménez, Marta Bjornson, Randi A Famula, Dominique D A Pincot, Michael A Hardigan, Mary A Madera, Cindy M Lopez Ramirez, Glenn S Cole, Mitchell J Feldmann, Steven J Knapp

**Affiliations:** Department of Plant Sciences, University of California, One Shields Avenue, Davis, California 95616, USA; Department of Plant Sciences, University of California, One Shields Avenue, Davis, California 95616, USA; Department of Plant Sciences, University of California, One Shields Avenue, Davis, California 95616, USA; Department of Plant Sciences, University of California, One Shields Avenue, Davis, California 95616, USA; Horticultural Crops Production and Genetic Improvement Research Unit, United States Department of Agriculture Agricultural Research Service, 3420 NW Orchard Avenue, Corvallis, Oregon 97330, USA; Department of Plant Sciences, University of California, One Shields Avenue, Davis, California 95616, USA; Department of Plant Sciences, University of California, One Shields Avenue, Davis, California 95616, USA; Department of Plant Sciences, University of California, One Shields Avenue, Davis, California 95616, USA; Department of Plant Sciences, University of California, One Shields Avenue, Davis, California 95616, USA; Department of Plant Sciences, University of California, One Shields Avenue, Davis, California 95616, USA

## Abstract

Wildtype fruit of cultivated strawberry (*Fragaria*  $\times$  *ananassa*) are typically soft and highly perishable when fully ripe. The development of firm-fruited cultivars by phenotypic selection has greatly increased shelf-life, decreased postharvest perishability, and driven the expansion of strawberry production worldwide. Hypotheses for the firm-fruited phenotype include mutations affecting the expression of genes encoding polygalacturonases (PGs) that soften fruit by degrading cell wall pectins. Here we show that loss-of-function mutations in the fruit softening gene *POLYGALACTURONASE1* (*FaPG1*; *PG1-6A1*) double fruit firmness in strawberry. *PG1-6A1* was one of three tandemly duplicated PG genes found to be in linkage disequilibrium (LD) with a quantitative trait locus (QTL) affecting fruit firmness on chromosome 6A. *PG1-6A1* was strongly expressed in soft-fruited (wildtype) homozygotes and weakly expressed in firm-fruited (mutant) homozygotes. Genome-wide association, quantitative trait transcript, DNA sequence, and expression-QTL analyses identified genetic variants in LD with *PG1-6A1* that were positively correlated with fruit firmness and negatively correlated with *PG1-6A1* expression. An *Enhancer/Suppressor-mutator* (*En/Spm*) transposable element insertion was discovered upstream of *PG1-6A1* in mutant homozygotes that we hypothesize transcriptionally downegulates the expression of *PG1-6A1*. The *PG1-6A1* locus was incompletely dominant and explained 26–76% of the genetic variance for fruit firmness among phenotypically diverse individuals. Additional loci are hypothesized to underlie the missing heritability. Highly accurate codominant genotyping assays were developed for modifying fruit firmness by marker-assisted selection of the *En/Spm* insertion and single nucleotide polymorphisms associated with the *PG1-6A1* locus.

## Introduction

The development of cultivars with increased fruit firmness and decreased perishability has been one of the most important domestication changes in cultivated strawberry (*Fragaria*  $\times$  *ananassa*), a naturally soft-fruited species [1–5]. Wildtype fruit are generally, but not universally, super soft and highly perishable when fully ripe, characteristics that enhance seed dispersal in nature [2, 6, 7]. Nevertheless, significant genetic variation for fruit firmness has been observed in strawberry, which has facilitated the development of cultivars with a wide range of fruit firmness and shelf-life characteristics [4, 6–8].

Strawberry is a nonclimacteric fruit that ripens gradually and lacks a well-defined climacteric peak, the point in the ripening process when ethylene production and respiration rate sharply increase in climacteric fruits [9–11]. That characteristic means that ripening ceases once the fruit are harvested and that flavor and texture are fixed at harvest and steadily deteriorate postharvest. The latter includes desiccation, the dissipation of volatile aromatic compounds, decreased glossiness, and rotting caused by gray mold (*Botrytis cinerea*) and other fungal diseases [10–17]. The speed of deterioration has long been known to be strongly correlated with the softness of the fruit at harvest [2, 9, 17–19].

The improvement of fruit firmness has a long history in strawberry breeding with references to cultivars developed in the late 1800s and early 1900s that were firmer than the wildtype [1, 2]. ‘Blakemore’, developed in 1931, was one of the earliest cultivars reported to “set the standard for firmness” necessary for shipping long distances without significant postharvest losses [3]. The firmness of that cultivar and other early firm-fruited cultivars, however, has been surpassed by the firmness of ‘Royal Royce’, ‘Eclipse’, and other long shelf-life (LSL) cultivars developed since the 1970s in California [2, 4, 5, 20]. Using soft-fruited wild relatives and heirloom cultivars as benchmarks, Feldmann et al. [5] showed that the genetic gains from traditional breeding for increased fruit firmness have been in the 240–770% range since the 1950s. The genetic factors underlying those genetic gains, however, are unknown, partly because the genetics of fruit firmness appears to be complex in strawberry [21–24]. Royce S. Bringhurst has been credited with identifying sources of favorable alleles for increased fruit firmness and developing the firm-fruited cultivars that revolutionized strawberry production in California [2, 4, 5], several of which were included in the genetic studies reported here.

**Figure 1:**
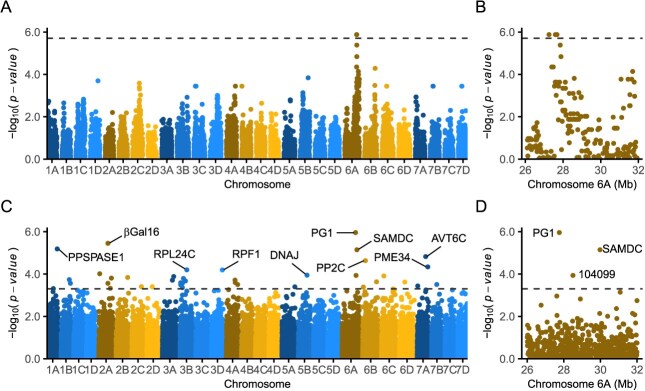
GWAS and QTT analyses identify genetic variants associated with phenotypic variation for fruit firmness and transcripts associated with differentially expressed genes among soft- and firm-fruited individuals ($n=\mathrm{85}$). Study population individuals were genotyped for 49 330 SNPs physically anchored to the FaRR1 reference genome. (A) The GWAS Manhattan plot illustrates SNPs associated with fruit firmness across the strawberry genome (physical positions of array-genotyped SNP on the *x*-axis are shown in the FaRR1 reference genome). GWAS was applied to phenotypic means estimated from 24 observations/individual using a Bonferroni-corrected significance threshold of 5.7 (depicted by the horizontal dashed line). (B) The physical positions of SNPs associated with phenotypic variation for fruit firmness are shown for Mb 26–32 on chromosome 6A. (C) The QTT Manhattan plot was constructed from analyses of 59 126 transcripts mapped in the FaRR1 reference genome using mRNAs isolated from ripe fruit of soft- or firm-fruited individuals in the study population ($n=\mathrm{85}$). QTT was applied to transcript counts estimated from short-read mRNA sequences using a Bonferroni-corrected significance threshold of 3.3 (depicted by the horizontal dashed line). The differentially expressed genes labeled in the QTT Manhattan plot are pyrophosphate-specific phosphatase1, $\beta$-galactosidase 16 ($\beta$Gal16), ribosomal protein L24C, RNA processing factor 1, chaperone DnaJ-domain, PG1, SAMDC, protein phosphatase 2c, pectin methylesterase 34, and amino acid transporter avt6c. (D) The physical positions of differentially expressed genes are shown for Mb 26–32 on chromosome 6A. Fxa6Ag104099 (abbreviated 104 099) is a gene of unknown function.

The phenotypic variation observed for fruit firmness in cultivated strawberry has been shown to be highly correlated with the expression of *POLYGALACTURONASE1* (*FaPG1*; abbreviated *PG1*), a fruit softening gene expressed in the fleshy receptacle [18, 25–27]. *PG1* plays an important role in pectin disassembly and fruit softening in the wildtype [25]. Villarreal et al. [18] showed that the expression of *PG1* was correlated with fruit firmness variation. The downregulation of *PG1* by antisense RNA silencing in transgenic plants and CRISPR/Cas9 insertion mutations of *PG1* in gene-edited plants either greatly decreased or eliminated the expression of *PG1* and increased fruit firmness [19, 25–30]. Paniagua et al. [19] showed that resistance to fungal decay and the speed of deterioration of the fruit was decreased by knocking out *PG1*.

Using gene annotations in the earliest assembly of the octoploid genome [31] and a phenotypically diverse genome-wide association study (GWAS) population, Hardigan et al. [32] discovered that three tandemly duplicated paralogs of *PG1* were in LD with a large-effect QTL for fruit firmness on chromosome 6A (labeled here using the chromosome nomenclature of Hardigan et al. [33]). They hypothesized that one of three paralogs was the causal gene underlying the QTL. Our study built on those findings. The QTL identified on chromosome 6A by Hardigan et al. [32] appears to be identical to a QTL identified in other forward genetic studies [21–24], a conclusion we reached by cross-referencing linkage group nomenclatures using the translation codex developed by Hardigan et al. [33].

By aligning the DNA sequence for the *FaPG1* gene (https://www.ncbi.nlm.nih.gov/nuccore/AF380299; [26, 27]) to the ‘Camarosa’ and ‘Royal Royce’ reference genomes and cross-referencing positions of physically-anchored array-genotyped single nucleotide polymorphisms (SNPs) ([31, 34]; https://phytozome-next.jgi.doe.gov/info/FxananassaRoyalRoyce_v1_0), we hypothesized that the fruit firmness QTL identified in previous forward genetic studies was likely caused by loss-of-function mutations affecting one of the three tandemly duplicated PG genes (*PG1-6A1*, −*6A2*, and −*6A3*) found to be in LD with the QTL on chromosome 6A [32]. We tested this and searched for additional genotype-to-phenotype associations using GWAS, transcript-to-phenotype associations using quantitative trait transcript (QTT) analysis, and genotype-to-transcript associations using expression-quantitative trait locus (eQTL) analysis [35–40].

Here we show that a loss-of-function mutation in one of the three genes (*PG1-6A1*, the paralog identified to be *FaPG1*) doubled fruit firmness in strawberry, that the *PG1-6A1* gene is strongly expressed in ripe fruit of wildtype homozygotes and weakly expressed in ripe fruit of mutant homozygotes, that the favorable (mutant) allele (*PG1-6A1*${}^{+}$) explains a significant fraction of the genetic variance for fruit firmness, and that firm-fruited cultivars are homozygous for the favorable allele (*PG1-6A1*${}^{+}$/*PG1-6A1*${}^{+}$). Although the causal mutation remains uncertain, we show that the mutant phenotype is associated with an *Enhancer/Suppressor-mutator* (*En/Spm*) transposable element (TE) insertion upstream of *PG1-6A1* and hypothesize that this TE disrupts the transcription of *PG1-6A1* in firm-fruited individuals [41]. Lastly, we describe highly predictive, high-throughput, codominant genotyping assays for marker-assisted selection (MAS) of genetic variants in LD with the *PG1-6A1* locus, and discuss the utility of native *PG1-6A1* alleles for modifying fruit firmness in strawberry.

## Results

### GWAS confirmed the segregation of a large-effect QTL for fruit firmness on chromosome 6A

To identify genetic variants associated with fruit firmness, we analyzed a population of 85 phenotyped, 50 K Axiom SNP array genotyped, and ripe-fruit transcriptome profiled hybrid individuals developed at the University of California, Davis (Supplemental Table S1 ). Their phenotypic means ($\bar{y}$) were estimated from multiple biological replicates (clones of hybrid individuals) and multiple harvests of ripe fruit over two growing seasons (24 phenotypic observations/individual), ranged from extremely soft (0.13 kg-force) to extremely firm (0.45 kg-force), and were approximately normally distributed (Supplemental Table S1 ). This population was dominated by firm-fruited individuals: 74 had phenotypic means in the 0.30 to 0.49 kg-force range (the firmest individual was 17C140P012). This group included the cultivar ‘Royal Royce’ ($\bar{y}=\mathrm{0.48}$ kg-force), 11 other UC cultivars, and 62 other UC hybrid individuals. The soft-fruited group included the UC cultivar ‘Chandler’ ($\bar{y}=\mathrm{0.21}$) and 10 other UC hybrid individuals. Approximately half of the phenotypic variation observed for fruit firmness in this discovery population was genetic. The REML estimates of narrow- and broad-sense heritability were ${h}^2=\mathrm{0.46}$ and ${H}^2=\mathrm{0.50}$, respectively; hence, 92% of the genetic variance for fruit firmness was estimated to be additive in the discovery population.

Using genetically validated physical positions of 49 330 array-genotyped SNPs for GWAS in the discovery population, four SNPs on chromosome 6A (AX-184953741, AX-184023221, AX-184726882, and AX-184210676) were found to be strongly associated with fruit firmness variation (Fig. 1A and B; Supplemental Tables S1 and S2). We did not observe statistically significant SNPs elsewhere in the genome, nor did [32] using a larger and more genetically diverse population ($n=\mathrm{460}$). We substantiated this by repeating the GWAS analysis of that population using the original phenotypic data and previously unpublished phenotypic data (10.9 fruit/individual $\times$ 460 individuals = 5014 phenotypic observations; Supplemental Table S1 ; https://datadryad.org/stash/share/IGQ-mlFL79PXSVBdGBRll-BovLCwf6CLxsa8egScqy4). The broad-sense heritability on a clone-mean basis was 0.81 for fruit firmness. The two most significant SNPs in our analysis of those data were AX-184107692 (−log${}_{10}$  *p*-value = 7.1; bp 27 812 192) and AX-184242253 (−log${}_{10}$  *p* value = 6.4; bp 27 888 596; Supplemental Fig. S1), both of which were in strong LD with SNPs identified in the discovery population ($n=\mathrm{85}$).

**Figure 2:**
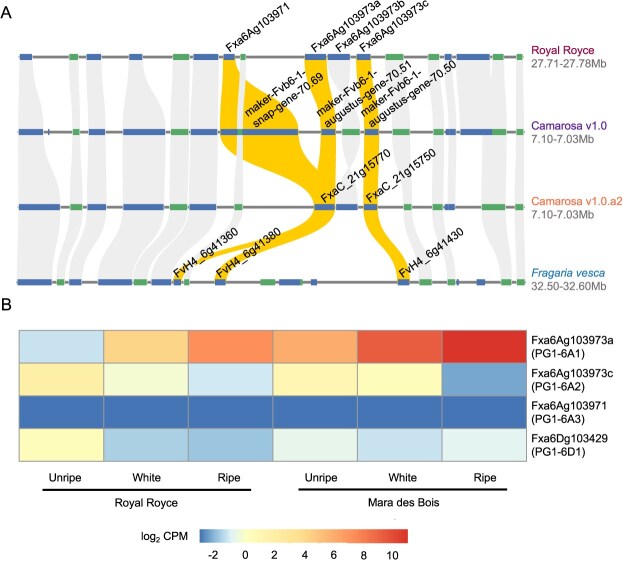
Annotations and physical positions of PG genes in LD with a fruit firmness QTL on chromosome 6A in octoploid strawberry. (A) Organization and synteny of three tandemly duplicated PG-encoding genes on chromosome 6A in the ‘Royal Royce’ and ‘Camarosa’ genomes and chromosome 6 in the ‘Hawaii 4’ *F. vesca* genome. (B) Transcript CPM for four PG-encoding genes observed in the soft-fruited cultivar ‘Mara des Bois’ and firm-fruited cultivar ‘Royal Royce’. CPMs were estimated from short-read RNA sequences normalized for sequencing depth.

**Figure 3:**
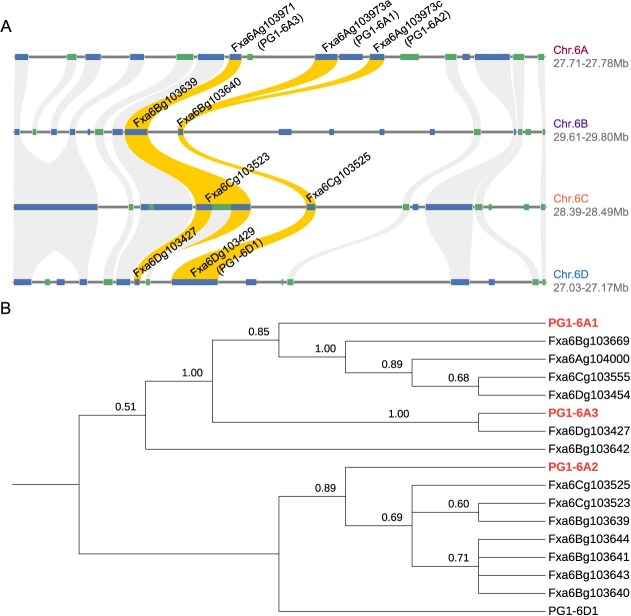
Local synteny and phylogenetic tree. A) Synteny analysis of homoeologous *PG1* genes across strawberry subgenomes using the ‘Royal Royce’ reference genome. Syntenic relationships among *PG1* genes in the four subgenomes are indicated by connecting lines. B) Evolutionary relationships among homoeologous *PG1* genes. The tandemly duplicated *PG1* genes found on chromosome 6A are shown in red. The tree was constructed using amino acid sequences of homoeologous *PG1* genes identified in the ‘Royal Royce’ genome. The numbers shown at nodes are bootstrap support for branches estimated from 1000 bootstrap samples.

**Figure 4:**
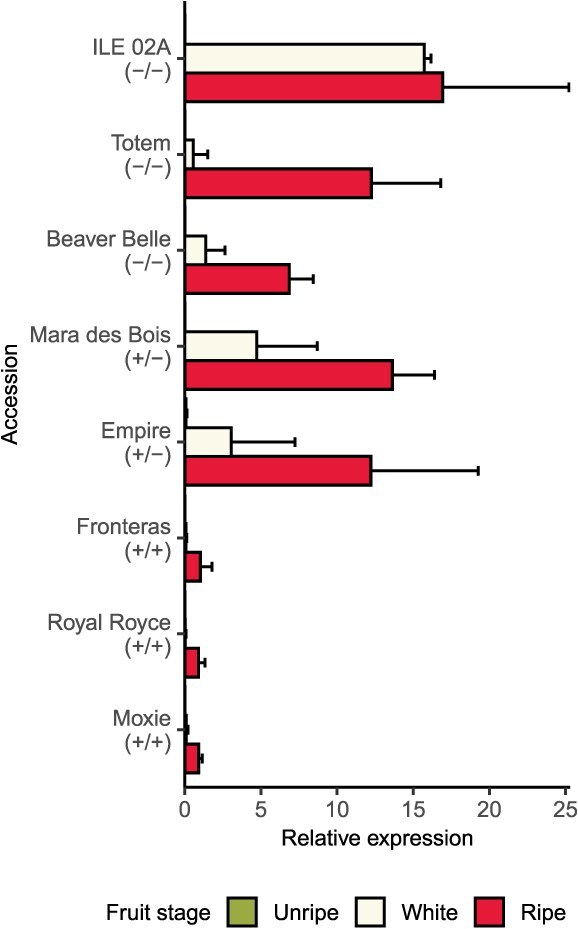
Quantitative RT-PCR analyses of *PG1-6A1* transcripts observed in unripe to ripe fruit of strawberry cultivars with different *PG1-6A1* genotypes and fruit firmness phenotypes. The *PG1-6A1* genotypes shown for each cultivar were predicted by genotypes of the *En/Spm* INDEL upstream of *PG1-6A1* and by phenotypic means for fruit firmness, where −/− are unfavorable allele homozygotes, +/− are heterozygotes, and +/+ are favorable allele homozygotes. The relative expression (RE) of *PG1-6A1* was estimated from analyses of three technical replicates/biological replicate $\times$ three biological replicates/stage/cultivar using a DNA-binding protein as a reference control. The bar depicts the mean, whereas the needle depicts one standard deviation from the mean. The bars and needles are barely perceptible in the unripe-green fruit stage for every *PG1-6A1* genotype and in the unripe-white fruit stage for mutant homozygotes (*PG1-6A1*^+^/*PG1-6A1*^+^) because the expression of *PG1-6A1* was exceptionally weak in those samples.

The four statistically significant SNPs identified by GWAS in the discovery population ($n=\mathrm{85}$) were in complete LD with one another ($\hat{r}^2=\mathrm{1.0}$), spanned a 0.6 Mb haploblock on chromosome 6A in the FaRR V1 reference genome (Mb 27.3–27.9 in FaRR V1; Supplemental Table S3), and exceeded a 1% false discovery rate (FDR)-corrected statistical significance threshold (Fig. 1). They were linked with array-genotyped SNPs previously shown to be associated with a fruit firmness QTL and three tandemly duplicated PG-encoding genes (Mb 7.046–7.064) on chromosome 6A [32]. The latter were annotated as FxaC_6-1 g13880, FxaC_6-1 g13900, and FxaC_6-1 g13910 in the ‘Camarosa’ reference genome (FaCA V1; [31]).

### Three tandemly duplicated *PG* genes are in strong LD with the fruit firmness QTL on chromosome 6A

Transcriptomic and BLAST analyses confirmed that the three PG-encoding genes in LD with the QTL on chromosome 6A are *FaPG1* (*PG1*) paralogs (Fig. 2A; [25–27]); however, we discovered errors in their annotations of the ‘Camarosa’ genome (FaCA V1; [31]), a reannotation of the ‘Camarosa’ genome (FaCA V1R; [42]), and annotations of the ‘Royal Royce’ genome (FaRR V1; https://phytozome-next.jgi.doe.gov/info/FxananassaRoyalRoyce_v1_0). Specifically and briefly, the first *PG1* paralog was merged with an *En/Spm* transposon and other nongenic DNA sequences in the original annotation of the ’Camarosa’ genome [31], the first *PG1* paralog was missing in the reannotation of the ‘Camarosa’ genome by Liu et al. [42], and the second and third *PG1* paralogs were merged with a gene of unknown function (Fxa6Ag103973) in the original annotation of the ‘Royal Royce’ genome (https://phytozome-next.jgi.doe.gov/info/FxananassaRoyalRoyce_v1_0). These annotation errors were manually corrected by mapping transcripts isolated from unripe and ripe fruit to the ‘Royal Royce’ reference genome (FaRR V1). We identified three tandemly duplicated *PG1* paralogs, which appear to be arranged as shown in the uppermost chromosome displayed in Fig. 2A. Fxa6Ag103973 in the original FaRR V1 annotation was split into two *PG1* paralogs (Fxa6Ag103973a and Fxa6Ag103973c) and a gene of unknown function (Fxa6Ag103973b) in the corrected annotation of FaRR V1.

The imperfections inherent in genome annotations [43], and the need to distinguish paralogs and homologs [44] underscore the complications that commonly arise when cross-referencing genes, loci, and alleles in genetic studies of octoploid strawberry, a species where community-wide guidelines have not yet been adopted for naming chromosomes, linkage groups, homologs and homoeologous genes, haplotypes, and alleles (Fig. 2A-Fig. 3). To address this in the present study, we created and adopted gene names using chromosome numbers (CNs) (1–7) and genome letters (A-D) as suffixes followed by consecutive numbers ($1,2,\dots, n$) to identify paralogs where necessary. The chromosome identifiers (1A, 1B, 1C, 1D, . . ., 7A, 7B, 7C, 7D) follow the nomenclature proposed by Hardigan et al. [33]. CNs (1, 2, . . ., 7) in the A, B, C, and D genomes are identical to those originally assigned in *F. vesca*, the diploid ancestor of the A genome [31, 45–47].

Using these naming guidelines and the corrected FaRR V1 gene annotations, the gene identifiers for the three tandemly duplicated *PG1* paralogs in LD with the fruit firmness QTL on chromosome 6A are *PG1-6A3* (Fxa6Ag103971), *PG1-6A1* (Fxa6Ag10397a), and *PG1-6A2* (Fxa6Ag10397c), as shown in Fig. 2A. Their arrangement and annotations match those found in the ‘Hawaii 4’ reference genome for *F. vesca* (FvH4 V1; [47]), as shown in the lowermost chromosome displayed in Fig. 2A. The sequences of these genes in FaRR V1 are 99.5% identical to their homologs in the FvH4 V1 diploid and 100.0% identical to their homologs in the FaCA V1 octoploid reference genomes. *PG1-6A1* is 73.9% identical to *PG1-6A2* and 61.5% identical to *PG1-6A3* (E $\le 1.0\times{10}^{-43}$). The *PG1-6A1* sequence in the UC cultivar ‘Royal Royce’ is 100% identical to the *FaPG1* sequence previously isolated from the UC cultivar ‘Chandler’ (https://www.ncbi.nlm.nih.gov/nuccore/AF380299; [26, 27]).

### A single paralog of the fruit softening gene *POLYGALACTURONASE1* underlies the fruit firmness QTL on chromosome 6A

We hypothesized that one of the three tandemly duplicated *PG1* paralogs was the causal gene underlying the fruit firmness QTL on chromosome 6A (Fig. 2). To test this, QTT analysis was applied to short-read sequences of ripe-fruit RNAs isolated from the discovery population (Fig. 1B; Supplemental Tables S1 and S4; https://www.ncbi.nlm.nih.gov/bioproject/PRJNA787565). Transcripts were observed in ripe fruit for 1081 of the 1761 annotated genes found in the 2.4 Mb genomic segment flanking the QTL. Three genes (*PG1-6A1* = Fxa6Ag103973a, Fxa6Ag104099, and Fxa6Ag104340) were found to be significantly associated with the fruit firmness QTL (*p* ≤ 0.005; Fig. 1B; Supplemental Table S4). *PG1-6A1* was in complete LD with the four peak SNPs identified by GWAS in the discovery population (Fig. 1D). Moreover, the expression of *PG1-6A1* was negatively genetically correlated with the firmness of ripe fruit ($r=-0.47$; *p* = 1 × 10^−6^) in the discovery population (Fig. 1; Supplemental Table S1 ). The function of Fxa6Ag104099 remains unknown, while the other QTT-significant gene (Fxa6Ag104340) encodes an S-adenosyl-L-Met decarboxylase gene (*FaSAMDC*) known to play an important role in the regulation of strawberry fruit ripening [48]. *FaSAMDC* is located approximately one Mb downstream of *PG1-6A2*; hence, we concluded that the fruit firmness variation associated with the QTL on chromosome 6A was likely caused by the segregation of mutant and wildtype *PG1-6A1* alleles (Fig. 1; Supplemental Table S4). Throughout this paper, alleles that increase fruit firmness are described as favorable and identified by a plus sign superscript (*PG1-6A1*^+^), whereas alleles that decrease fruit firmness are described as unfavorable and identified by a minus sign superscript (*PG1-6A1*^−^). These designations are market class dependent, e.g., mutant alleles that knockdown or knockout the expression of *PG1-6A1* are favorable when breeding firm-fruited cultivars and unfavorable when breeding soft-fruited cultivars.

Our BLAST analyses suggest that approximately 227 PG-encoding genes have survived polyploid evolution in the octoploid (compiled in Supplemental Table S5). Apart from the three *PG1* paralogs on chromosome 6A, 14 *PG1* homoeologs were identified on chromosomes 6B, C, and D (Fig. 3; Supplemental Table S5). Of these, only *PG1-6A1* was strongly expressed in ripe fruit (Fig. 2). *PG1-6A2*, *PG1-6A3*, and Fxa6Dg103429 (*PG1-6D1*), a *PG1* homolog on chromosome 6D, were very weakly expressed in unripe-green fruit and unexpressed in ripe fruit (Fig. 2B). The other 13 *PG1* B-, C-, and D-genome homoeologs were unexpressed in fruit (Supplemental Fig. S2).

The transcriptomes of the soft-fruited cultivar ‘Mara des Bois’ ($\overline{y}$ = 0.10 kg-force) and firm-fruited cultivar ‘Royal Royce’ ($\overline{y}$ = 0.35 kg-force) were compared by RNA-seq across different stages of fruit development, where $\overline{y}$ is the estimated marginal mean (EMM) for fruit firmness (Fig. 2B). As predicted by our QTT analysis, *PG1-6A1* was more strongly expressed in ripe fruit of the soft-fruited wildtype (‘Mara des Bois’; 2033.1 CPM) than the firm-fruited mutant (‘Royal Royce’; 163.2 CPM), a 12.5-fold difference (Fig. 2B). The expression of *PG1-6A1* increased from 57.0 CPM in unripe to 2033.1 CPM in ripe fruit of the wildtype (a 35.7-fold increase), and from 0.3 CPM in unripe to 163.2 CPM in ripe fruit of the mutant (a 544.0-fold increase). The weak *PG1-6A1* expression in ripe fruit of the firm-fruited cultivar ‘Royal Royce’ was consistent with the observation in reverse genetic studies that *PG1* knockdown and knockout mutations result in increased fruit firmness [25, 27].

To further substantiate the association between *PG1-6A1* expression and the QTL, we developed paralog-specific quantitative reverse transcription polymerase chain reaction (qRT-PCR) assays for *PG1-6A1*, *PG1-6A2*, and *PG1-6D1* and screened RNAs isolated from unripe, green, and ripe fruit of three firm-fruited and five soft-fruited cultivars (Fig. 4; Supplemental Fig. S3). *PG1-6A1*, *PG1-6A2*, and *PG1-6D1* are the only *PG1* paralogs that were observed to be weakly to strongly express in developing fruit (Supplemental Fig. S2). The expression of *PG1-6A1* was weak in unripe-green fruit of every cultivar, progressively increased in the five soft-fruited cultivars as they ripened, was 13.0-fold greater in ripe fruit of soft-fruited than firm-fruited cultivars, and was negatively correlated with fruit firmness in ripe fruit ($\hat{r}=-0.82$; $p = 1.4\times{10}^{-2}$; Fig. 4). Our analyses substantiated that *PG1-6A2* and *PG1-6D1* were very weakly expressed in developing fruit and that their expression was uncorrelated with fruit firmness variation (Supplemental Fig. S3).

**Table 1:** Statistics^a^ for an *En/Spm* INDEL and SNPs associated with a *PG1-6A1* and fruit firmness QTL on chromosome 6A in a population of 43 soft- to firm-fruited individuals.

		Position						$\hat{a}$		$\hat{d}$		
Marker (M)^b^	Variant^c^	(bp)	FAF^d^	${\hat{r}}_{\overline{y},M}$	${\hat{r}}_{RE,M}$	PVE	GVE	(kg-force)	$\mathit{\Pr}>$ *F*	(kg-force)	$\mathit{\Pr}>$ F	$\mid \hat{d}/\hat{a}\mid$
AX-184953741	G/T	27 253 734	0.49	0.77	−0.86	0.68	0.76	0.08	$<\mathrm{0.0001}$	0.04	0.07	0.48
AX-184210676	C/A	27 676 285	0.55	0.68	−0.91	0.22	0.26	0.07	$<\mathrm{0.0001}$	0.06	0.01	0.82
*En/Spm* INDEL	+/−	27 743 085	0.55	0.76	−0.79	0.49	0.55	0.08	$<\mathrm{0.0001}$	0.05	0.01	0.59
5′-UTR	G/T	27 751 732	0.45	0.81	−0.91	0.53	0.61	0.08	$<\mathrm{0.0001}$	0.03	0.24	0.33
AX-184242253	G/A	27 888 596	0.49	0.78	−0.75	0.54	0.61	0.08	$<\mathrm{0.0001}$	0.04	0.05	0.50

[a] Correlation between the phenotypic mean for fruit firmness ($\bar{y}$) and marker locus genotypes (${\hat{r}}_{\bar{y},M}$), correlation between the relative expression (RE) of *PG1-6A1* in ripe fruit and marker locus genotypes (${\hat{r}}_{RE,M}$), estimates of the additive ($\hat{a}$) and dominance (${\hat{d}}$) effects of the marker locus on fruit firmness, degree-of-dominance ($\mid{\hat{d}}/{\hat{a}}\mid$), fraction of the phenotypic variance explained by the marker locus (PVE), and fraction of the genetic variance explained by the marker locus (GVE). Correlation coefficient estimates were significantly greater than zero for every genetic variant (*p* $\le 0.0001$ for ${\hat{r}}_{\bar{y},M}$ and $0.008\le p\le 0.020$ for ${\hat{r}}_{RE,M}$). Additive and dominance effects were estimated by linear contrasts among genotypic means (${\bar{y}}_{+/+}$, ${\bar{y}}_{+/-}$, and ${\bar{y}}_{-/-}$). Significance levels ($\mathit{\Pr}>$ F) are shown in the columns to the right of additive and dominance effect estimates for tests of the null hypothesis that the linear contrast was not significantly different from zero, where ${\hat{a}}=\big({\bar{y}}_{+/+}-{\bar{y}}_{-/-}\mathrm{\big)/2}$ and ${\hat{d}}=\big({\bar{y}}_{+/+}+{\bar{y}}_{-/-}\mathrm{\big)/2}-{\bar{y}}_{+/-}$.

[b] Genetic variants were genotyped by GBS. SNP and INDEL genotypes were called by aligning long-read DNA sequences for 43 individuals to the ‘Royal Royce’ reference genome (FaRR1). AX-184953741 is one of four 50 K array SNP markers identified by GWAS that was most strongly associated with the QTL on chromosome 6A (see Supplemental Table S6). AX-184210676 is the 50 K array SNP marker identified by GWAS that was most tightly linked to *PG1-6A1* among the four that were strongly associated with the QTL on chromosome 6A and in complete LD within the discovery population (Supplemental Table S2). The 4948-bp *En/Spm* INDEL is located 3926 bp upstream of *PG1-6A1*. The G/T SNP in the 5′-UTR is one of three that were in complete LD in the 5′-UTR of *PG1-6A1*. Statistics were identical for the three highly predictive 5′-UTR SNPs identified by sequence and QTL analyses among the 43 DNA sequenced individuals and are only shown for the G/T SNP (bp 27 751 732). The other two 5′-UTR SNPS were A/G (bp 27 751 041) and T/C (bp 27 751 106). AX-184242253 is the 50 K array SNP in LD with *PG1-6A1* that was identified by expression-QTL analysis (Supplemental Table S7).

[c] The first nucleotide shown for each SNP is the favorable allele (the SNP associated with the *PG1-6A1* allele predicted to increase fruit firmness.) The 4498-bp insertion (+) is the favorable allele for the INDEL.

[d] FAF is the frequency of the SNP or *En/Spm* INDEL allele associated with the favorable *PG1-6A1* allele.

**Figure 5:**
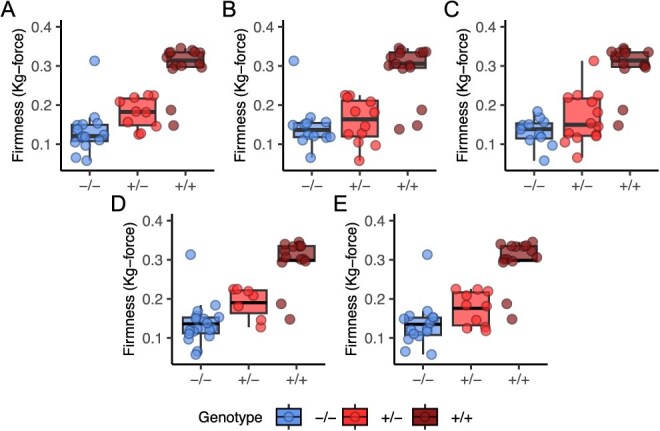
Fruit firmness variation among 43 soft- to firm-fruited individuals genotyped for an *En/Spm* INDEL and SNPs associated with the *PG1-6A1* locus on chromosome 6A. Genetic variants were genotyped using GBS. The points display phenotypic means (EMMs) estimated from five biological replicates (clones)/individual, five harvests, and three subsamples/replicate/harvest among greenhouse grown plants of the DNA sequenced individuals (11 observations/individual). The box displays the genotypic median and interquartile range within each genotypic class, where −/− are unfavorable allele homozygotes, +/− are heterozygotes, and +/+ are favorable allele homozygotes. (A) SNP interrogated by AX-184953741, one of four Axiom 50 K array SNP markers identified by GWAS found upstream of *PG1-6A1* and in complete LD with one another. (B) SNP interrogated by AX-184210676, one of four Axiom 50 K array SNP markers identified by GWAS found upstream of *PG1-6A1* and in complete LD with one another. (C) A 4948-bp *En/Spm* INDEL 3926 bp upstream of *PG1-6A1*. (D) A G/T SNP in the 5′-UTR of *PG1-6A1*. (E) SNP interrogated by AX-184242253, an Axiom 50 K array SNP marker identified by expression-QTL analysis found downstream of *PG1-6A1.*

### An *En*/*Spm* transposon insertion–deletion and SNPs associated with differentially expressed *PG1-6A1* alleles

To search for prospective causal mutations and identify predictive genetic variants within and proximal to the *PG1-6A1* gene, we developed and analyzed high fidelity (long-read) DNA sequences for 43 individuals, including 37 *F.*  $\times$  *ananassa* cultivars, two *Fragaria chiloensis* ecotypes, and four *F. virginiana* ecotypes differing in fruit firmness (phenotypic means for these individuals ranged from 0.06 to 0.35 kg-force in a greenhouse study and 0.07 to 0.41 kg-force in field studies; Supplemental Table S1 ). DNA sequences for those individuals were aligned to the FaRR V1 reference genome to identify structural variants and SNPs associated with the *PG1-6A1* locus (Table 1; Fig. 5; Supplemental Fig. S4). Our analysis of the 0.6 Mb segment flanking the QTL (Mb 27.3–27.9) identified 7221 SNPs (one SNP every 83.1 bp) among the 43 individuals. The Mb 27.5–27.8 segment harboring the QTL and tandemly duplicated PG genes (*PG1-6A1*, *PG1-6A2*, and *PG1-6A3*) was homozygous in ‘Royal Royce’ and the other nine firm-fruited UC cultivars we sequenced (Supplemental Table S1 ). Analyses of pedigree records suggested that the favorable *PG1-6A1* alleles found in these cultivars are identical-by-descent [49].

We did not identify a definitive causal mutation in *PG1-6A1*; however, several genetic variants were found to be highly predictive of *PG1-6A1* expression differences and fruit firmness variation (Table 1; Fig. 5). Sorting intolerant from tolerant (SIFT) statistics estimated with SNPeff [50] and SNPsift [51] were used to search for SNPs predicted to affect the function of the PG encoded by *PG1-6A1*, especially nonsynonymous SNPs that could cause amino acid substitutions predicted to be deleterious to protein function (Supplemental Table S8). SIFT analyses were done by comparing *PG1-6A1* alleles between soft-fruited ($\bar{y}<\mathrm{0.30}$ kg-force) and firm-fruited ($\bar{y}\ge 0.30$ kg-force) individuals. Thirty-seven SNPs were identified among *PG1-6A1* alleles, 19 in the 5′-UTR, 12 in exons, four in introns, and two in the 3′-UTR (Supplemental Table S8; Supplemental Fig. S4). Of the 12 SNPs found in exons, four caused synonymous and eight caused nonsynonymous amino acid changes, none of which were predicted to be deleterious.

We used SIFT statistics and single-marker QTL analyses to identify genetic variants (SNPs and insertion–deletions [INDELs]) associated with fruit firmness phenotypes among the 43 long-read sequenced individuals (Table 1; Fig. 5; Supplemental Table S8). Three SNPs in complete LD with one another in the 5′-UTR (bp 27 751 041, 27 751 106, and 27 751 732; MAF = 0.45) were found to be positively correlated with fruit firmness ($\hat{r} =\mathrm{0.81}$; $p=\mathrm{1.73}\times{10}^{-8}$) and negatively correlated with the expression of *PG1-6A1* in ripe fruit ($\hat{r}=-0.91$; $p=\mathrm{0.002}$; data are only shown for one of the three in Table 2; Supplemental Table S8). A single exonic SNP (bp 27 752 865; MAF = 0.44) was similarly predictive of fruit firmness variation ($\hat{r}=\mathrm{0.76}$; $p=\mathrm{8.63}\times{10}^{-7}$). This SNP caused a conservative, nonsynonymous amino acid change (M220T) and was predicted by SNPsift to have a moderate effect on protein function, if any (Supplemental Table S8).

To complement these analyses, we used expression-QTL (eQTL) analysis to search the genome for associations between 50 K array genotyped SNPs and transcript abundance (count per million) in ripe fruit of the 85 discovery population individuals (Supplemental Tables S1 and S7). That analysis identified 10 780 eQTL associations in 5159 differentially expressed genes in ripe fruit transcriptomes (Supplemental Table S7). We identified a SNP (AX-184242253; *p* = 4.69 ×10^−10^) slightly downstream of *PG1-6A1* that was significant in our GWAS analysis, tightly linked with *PG1-6A1* and the four GWAS peak SNPs, positively correlated with variation in fruit firmness, and negatively correlated with *PG1-6A1* expression differences (Table 1; Fig. 1).

Our analyses of genotyping-by-sequencing (GBS)-called variants uncovered a 4948-bp INDEL 3926 bp upstream of *PG1-6A1* that was positively associated with fruit firmness variation ($\hat{r} =\mathrm{0.76}$; *p* < 0.0001) and negatively correlated with the expression of *PG1-6A1* in ripe fruit ($\hat{r}=-0.79$; *p* value = 0.02) (Fig. 4 and 5C; Table 1; Supplemental Fig. S4). The *En*/*Spm* insertion was only found in firm-fruited individuals, was homozygous in ‘Royal Royce’ and the other nine modern, firm-fruited UC cultivars that were long-read sequenced, and was the only structural variant proximal to *PG1-6A1* that was strongly associated with variation in fruit firmness and expression of *PG1-6A1* in wildtype and mutant individuals (Table 1; Fig. 4; Supplemental Table S1 ). The 4948-bp insertion was discovered to be a CACTA family class II *En*/*Spm* TE with sequence similarity to *F. vesca*, *F.*  $\times$  *ananassa*, and other *En*/*Spm* TEs in plants [52–55]. This TE carries the characteristic 12-bp terminal inverted repeats (5$^{\prime}$-CACTACCAGAAA-3$^{\prime}$) and proximal subterminal direct repeats found in CACTA family class II TEs [52, 56, 57].

**Figure 6:**
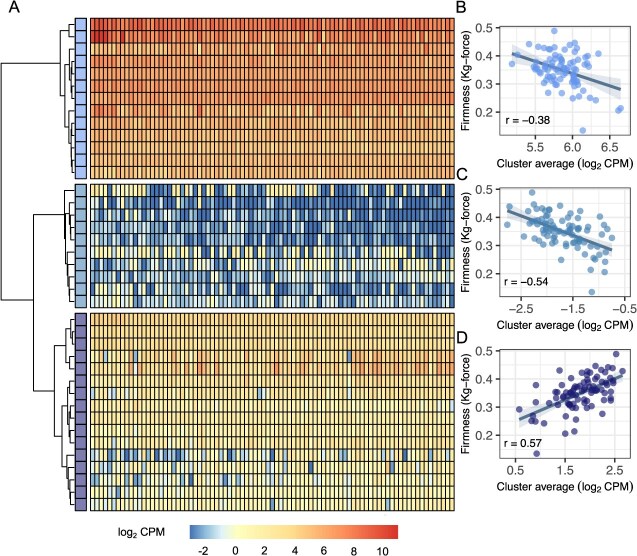
Co-expression network analysis of transcripts in ripe fruit of 85 discovery population individuals. (A) Transcript abundance heat map for genes in the *PG1-6A1* PG co-expression network (upper panel) and two other networks identified by co-expression analysis (middle and lower panels) using hierarchical cluster analysis. (B) The correlation between transcript abundance and fruit firmness for genes in the co-expression network is shown in the upper panel of A. (C) The correlation between transcript abundance and fruit firmness for genes (nodes) in the co-expression network is shown in the middle panel of A. (D) The correlation between transcript abundance and fruit firmness for genes (nodes) in the co-expression network is shown in the lower panel of A.

**Table 2:** The physical locations and putative functions of genes with statistically significant QTT analysis signals that belong to the *PG1-6A1* co-expression network.

Gene ID	CN	Position (bp)	Predicted function	Locus ID	*r*	*P* value
Fxa2Ag102750	2A	20 224 814-20 228 497	Proteasome $\beta$ subunit C1	AT1G21720	−0.39	$2.7\times{10}^{-4}$
Fxa2Ag102751	2A	20 233 768-20 239 168	$\beta$ -galactosidase 16	AT1G77410	−0.48	$3.5\times{10}^{-6}$
Fxa3Ag103343	3A	24 740 383-24 743 537	SEC14 phosphoglyceride transfer protein	AT3G46450	−0.40	$1.3\times{10}^{-4}$
Fxa3Bg200155	3B	726 016-729 670	Aspartic proteinase A1	AT1G11910	−0.38	$3.2\times{10}^{-4}$
Fxa3Bg200626	3B	3 688 029-3 691 371	Trehalose-6-phosphate synthase/phosphatase	AT1G06410	−0.37	$4.4\times{10}^{-4}$
Fxa3Bg200627	3B	3 696 159-3 701 280	Valine-tolerant 1	AT5G16290	−0.39	$2.7\times{10}^{-4}$
Fxa4Ag102677	4A	21 393 662-21 398 206	Protein phosphatase 2A subunit A2	AT3G25800	−0.39	$2.5\times{10}^{-4}$
Fxa6Ag103973a	6A	27 750 926-27 751 957	PG 1	AT3G07820	−0.50	$1.1\times{10}^{-6}$
Fxa6Ag104340	6A	29 976 026-29 976 574	S-adenosylmethionine decarboxylase	AT3G02470	−0.47	$7.2\times{10}^{-6}$
Fxa6Dg101561	6D	10 880 821-10 881 339	A20/AN1-like zinc finger protein	AT2G36320	−0.39	$2.4\times{10}^{-4}$
Fxa7Ag200163	7A	1 310 646-1 315 814	Sterol methyltransferase 1	AT5G13710	−0.38	$3.6\times{10}^{-4}$
Fxa7Ag203101	7A	21 166 929-21 170 332	Pectin methylesterase 34	AT3G49220	−0.43	$4.6\times{10}^{-5}$
Fxa7Bg201670	7B	14 335 172-14 342 432	SET domain protein 14	AT3G61740	−0.38	$3.1\times{10}^{-4}$

The correlation (*r*) between transcript abundance (count per million) and fruit firmness was estimated from short-read RNA sequences of 85 individuals. CNs, physical positions, and gene IDs are from annotations of genes in the Royal Royce reference genome. The predicted gene functions and Arabidopsis Genome Initiative locus IDs are from annotations of genes in The Arabidopsis Information Resource (width = 1tw).

### Wildtype *PG1-6A1* alleles are incompletely dominant

Table 1 displays the additive and dominance effects and other statistics for the five GBS-called genetic variants that were most highly predictive of *PG1-6A1* expression differences and fruit firmness variation in diverse germplasm (Supplemental Table S1 ). These include array-genotyped SNPs identified by GWAS (AX-184953741 and AX-184210676) or expression-QTL analysis (AX-184242253), one of three SNPs in the 5′-UTR of *PG1-6A1* identified by SIFT, sequence, and QTL analyses, and the *En/Spm* INDEL identified by sequence and QTL analyses (Table 1; Fig. 5; Supplemental Table S7). The three SNPs in the 5′-UTR of *PG1-6A1* were in complete LD among the 43 long-read sequenced individuals; hence, the statistics shown for the G/T SNP (bp 27 751 732) were identical for the other two SNPs among those 43 individuals (Table 1).

The favorable allele frequencies, correlations between marker genotypes and phenotypic means (${\hat{r}}_{\bar{y},M}$), correlations between marker genotypes and relative expression (RE) levels of *PG1-6A1* (${\hat{r}}_{\bar{y},M}$), and additive (${\hat{a}}$) and dominance (${\hat{d}}$) effect estimates were similar across genetic variants (Table 1; Fig. 5). The additive effects of these *PG1-6A1*-associated genetic variants were highly significant (*p* ≤ 0.0001) and greater than their dominance effects, which were significant for three of the five genetic variants (*p* ≤ 0.05). The wildtype *PG1-6A1* allele was incompletely dominant to nearly dominant. Our ($\mid{\hat{d}}/{\hat{a}}\mid$ estimates ranged from 0.33 to 0.82 for the five genetic variants (Table 1; Fig. 5). Four of the genetic variants explained 49-68% of the phenotypic variance and 55-76% of the genetic variance for fruit firmness among the 43 long-read sequenced individuals. Our estimate of the genetic variance explained by the AX-184210676 SNP (26%) in the population of 43 long-read-sequenced individuals was markedly lower than estimates for the other genetic variants, even though the *PG1-6A1* genotypes predicted by that SNP were strongly correlated with differences in *PG1-6A1* expression and phenotypic variation for firm firmness in the discovery population (Table 1).

### QTT and co-expression analyses identified several differentially expressed genes known or predicted to affect fruit development and ripening

We knew from GWAS analyses that approximately 24-45% of the heritability for fruit firmness was likely missing and undoubtedly caused by the segregation of loci other than *PG1-6A1* in the discovery population (Table 1). Although we only observed one statistically significant genotype-to-phenotype QTL in the GWAS, multiple QTL have been identified in other forward genetic studies [21, 22, 58], and several statistically significant transcript-to-phenotype QTLs were observed in our QTT analysis (Fig. 1). To shed light on the biological functions of the latter, we conducted a co-expression analysis of the 39 genes identified by QTT analysis (Fig. 6; Table 2; Supplemental Table S4). This revealed three co-expression modules: one comprised of genes that were moderately to strongly expressed in ripe fruit and negatively correlated with fruit firmness (module 1, upper clade); one comprised of genes with low expression in ripe fruit that were negatively correlated with fruit firmness (module 2, middle clade); and one comprised of genes with that were moderately to strongly expressed in ripe fruit and positively correlated with fruit firmness (module 3, lower clade), as shown in Fig. 6. These three modules contain genes with documented connections to fruit ripening and development that warrant further study (Table 2; Supplemental Table S4).

Notably, module 1 contains *PG1-6A1*, *FaSAMDC* [48], and other genes involved in cell wall remodeling, e.g., $\beta$-galactosidase 16 [26, 59] and pectin methylesterase 34 [60]. Although the genes in module 2 were less strongly expressed than those in module 1, several were identified to have functions associated with abscisic acid signaling through phosphate metabolism [61, 62] and fruit softening through carbon metabolism and methylation reactions e.g., a glycogenin-like starch initiation protein [63] and a SAM-dependent methyltransferase [63–65]. Interestingly, module 3 contains several genes with DNA/RNA binding activity, which may reflect the alternate metabolic profile of firm fruit with reduced cell wall breakdown. For example, the restorer of fertility-like pentatricopeptide repeat protein is essential for efficiently processing a mitochondrial NAD4 mRNA [66]. Incorrect splicing of the mitochondrial NAD4 transcript might disrupt carbon metabolism and ATP production due to abnormal NADH metabolism [67]. Further study of these genes, some of which have not been studied or well characterized, could shed additional light on the processes underlying fruit softening in strawberry.

**Table 3:** Statistics^a^ for KASP markers^b^ developed for genotyping an *En/Spm* INDEL and SNPs associated with the *PG1-6A1* locus on chromosome 6A.

	Reference	KASP		Accuracy	Call rate		$\hat{a}$		$\hat{d}$		
Population^c^	Variant	Marker (M)	FAF^d^	(%)^e^	(%)^f^	${\hat{r}}_{\hat{y},M}$	(kg-force)	$\mathit{\Pr}>$ F	(kg-force)	$\mathit{\Pr}>$ F	$\mid \hat{d}/\hat{a}\mid$
Diversity	AX-184210676	K-676	0.55	97.8	98.9	0.67	0.09	$<\mathrm{0.0001}$	0.04	0.05	0.44
	*En/Spm* INDEL	K-SPM	0.65	81.0	86.0	0.64	0.09	$<\mathrm{0.0001}$	0.00	0.95	0.02
	5′-UTR	K-732	0.55	100.0	82.8	0.82	0.10	$<\mathrm{0.0001}$	0.01	0.38	0.14
	AX-184242253	K-253	0.50	95.6	100.0	0.83	0.10	$<\mathrm{0.0001}$	0.02	0.22	0.19
Full sibs	AX-184210676	K-676	0.54	NA	99.4	0.35	0.04	$<\mathrm{0.0001}$	0.01	0.28	0.33
	*En/Spm* INDEL	K-SPM	0.62	NA	97.0	0.45	0.04	$<\mathrm{0.0001}$	0.03	0.01	0.74
	5′-UTR	K-732	0.40	NA	84.9	0.48	0.05	$<\mathrm{0.0001}$	0.03	0.01	0.56
	AX-184242253	K-253	0.50	NA	95.8	0.51	0.05	$<\mathrm{0.0001}$	0.02	0.03	0.41

[a] ${\hat{r}}_{\bar{y},M}$ is the correlation between the phenotypic mean ($\bar{y}$) for fruit firmness (kg-force) and marker genotypes, $({\hat{a}})$ is the additive and (${\hat{d}}$) is the dominance effect, and $\mid{\hat{d}}/{\hat{a}}\mid$ is the degree-of-dominance of the KASP marker locus. The correlation coefficient estimates (${\hat{r}}_{\bar{y},M}$) for every marker locus were highly significant (*p* ≤ 0.0001) in both populations. Additive and dominance effects were estimated by linear contrasts among genotypic means (${\bar{y}}_{+/+}$, $\bar{y}_{+/-}$, and ${\bar{y}}_{-/-}$). Significance levels ($\mathit{\Pr}>$  *F*) are shown in columns to the right of additive and dominance effect estimates for tests of the null hypothesis that the linear contrast was not significantly different from zero, where ${\hat{a}}=\big({\bar{y}}_{+/+}-{\bar{y}}_{-/-}\mathrm{\big)/2}$ and ${\hat{d}}=\big({\bar{y}}_{+/+}+{\bar{y}}_{-/-}\mathrm{\big)/2}-{\bar{y}}_{+/-}$.

[b] KASP markers were designed for the *En/Spm* INDEL and SNPs shown in Table 2.

[c] The diversity population consisted of 92 soft- to firm-fruited individuals. The full-sib population consisted of 152 one-year-old individuals within four full-sib families segregating for mutant and wildtype *PG1-6A1* alleles.

[d] FAF is the frequency of the KASP-SNP or KASP-INDEL marker allele associated with the favorable *PG1-6A1* allele.

[e] Accuracy (%) was estimated for the K-SPM and K-732 marker by comparing genotypes called by GBS among long-read DNA sequences of 43 individuals with genotypes called by KASP. Accuracy was estimated for the K-676 and K-253 markers by comparing genotypes called among 92 Axiom 50 K array-genotyped individuals with genotypes called by KASP. KASP marker accuracy could not be estimated among full-sib individuals because they were only genotyped with the KASP markers and were not sequenced or genotyped with the 50 K SNP array.

[f] The call rate is the percentage of individuals where KASP genotypes were successfully called.

**Figure 7:**
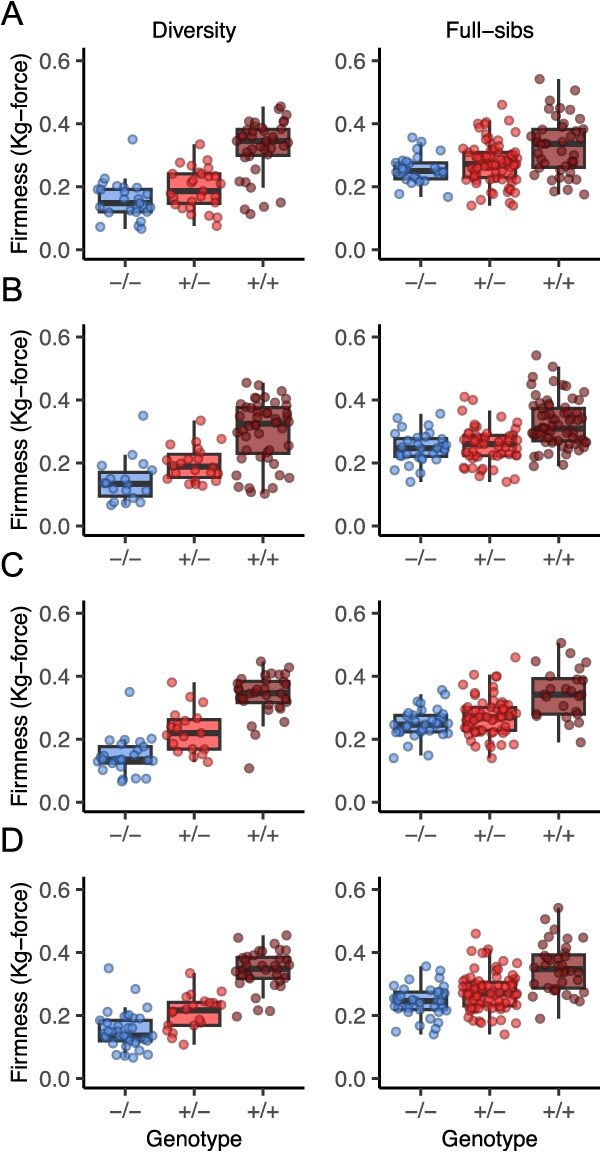
Fruit firmness variation among 92 soft- to firm-fruited individuals (the diversity population; left column) and 152 full-sib progeny (the full-sib population; right column) genotyped with KASP markers developed for an *En/Spm* INDEL and SNPs associated with the *PG1-6A1*) locus. The points display phenotypic means (EMMs) for 92 individuals in the diversity population (four observations/individual) and 152 individuals in the full-sib population (six observations/individual). The box displays the genotypic median and interquartile range within each genotypic class for each KASP marker, where −/− are unfavorable allele homozygotes, +/− are heterozygotes, and +/+ are favorable allele homozygotes. Genotypes and phenotypes are shown for four KASP markers associated with the *PG1-6A1 locus*: (A) K-676 (bp 27 676 285), K-SPM (bp 27 743 085), K-732 (bp 27 751 732), and K-253 (bp 27 888 596).

### Kompetitive allele-specific PCR **assays for** MAS **of**  *PG1-6A1***-associated genetic variants are specific and accurate**

To facilitate MAS of the *PG1-6A1* locus, Kompetitive Allele-Specific PCR (KASP) assays were developed and tested for one of the peak SNPs identified by GWAS (AX-184210676), three SNPs in the 5′-UTR identified by SIFT and QTL analyses, a SNP downstream of *PG1-6A1* identified by e-QTL analysis (AX-184242253), and the *En/Spm* INDEL (Table 3; Fig. 7; Supplemental Fig. S5; Supplemental Tables S1 and S9). To assess their specificity, accuracy, and predictive values, 92 individuals spanning the domestication and fruit firmness spectra in strawberry (hereafter the ‘diversity’ population) were selected and genotyped. They included 29 heirloom cultivars (1854–1991), 27 UC cultivars (1935–2017), 23 additional UC hybrid individuals (1961–2020), four *F. chiloensis* ecotypes, eight *F. virginiana* ecotypes, and a single *F. vesca* ecotype. Their fruit firmness phenotypes ranged from 0.07 to 0.45 kg-force (Supplemental Table S1 ).

**Figure 8:**
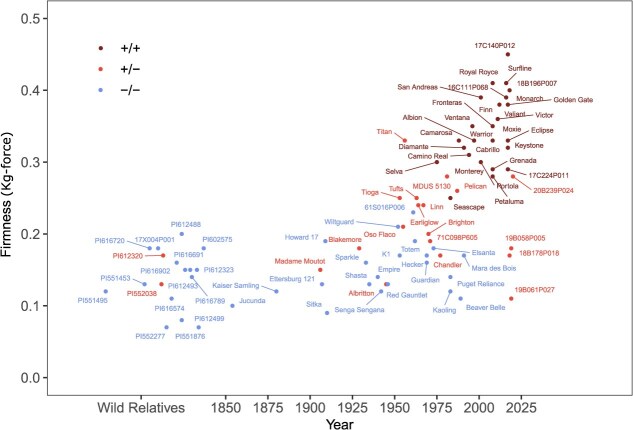
Fruit firmness phenotypes of wild relatives, cultivars, and other genetic resources of cultivated strawberry originating 1850 to present. The birth years of *F.*  $\times$  *ananassa* cultivars are plotted on the *x*-axis. The phenotypes of several *Fragaria chiloensis* and *F. virginiana* ecotypes are shown in random order to the left of 1850 on the *x*-axis. Genotypes of the AX-184242253 SNP were used to predict to *PG1-6A1* unfavorable allele homozygotes (−/−; blue points), heterozygotes (+/−; red points), and favorable allele homozygotes (+/+; brown points), where the favorable (mutant) allele (*PG1-6A2*${}^{+}$) increases fruit firmness. See Supplemental Fig. S6 for a version of this figure showing additional cultivars.

**Figure 9:**
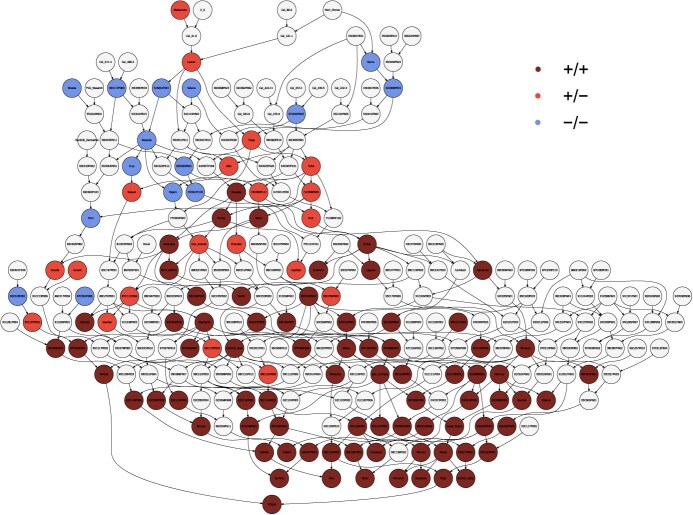
Tracing the ancestry of the favorable (mutant) *PG1-6A1* allele found in firm-fruited UC cultivars. The family tree illustrates a small fraction of the thousands of descendants of ‘Tioga’ and ‘Tufts’ in the pedigree records of firm-fruited progeny developed at UC, including every UC cultivar developed since 1970. Genotypes of the AX-184242253 SNP were used to predict *PG1-6A1* unfavorable allele homozygotes (−/−; blue points), heterozygotes (+/−; red points), and favorable allele homozygotes (+/+; brown points), where the favorable (mutant) allele (*PG1-6A2*${}^{+}$) increases fruit firmness. Gray nodes identify individuals that were not genotyped or phenotyped, many of which are extinct.

The KASP assays we developed for *PG1-6A1*-associated genetic variants appear to be paralog-specific (only amplified *PG1-6A1* alleles), but differed in genotyping accuracy and genotype call rates (Table 3; Fig. 7; Supplemental Fig. S5). Assays designed from the 50 K array SNPs (K-676 for the AX-184210676 SNP and K-253 for the AX-19424253 SNP) were highly accurate with distinct, codominant genotypic clusters and 98.9–100.0% genotype call rates (Table 3; Figs 1 and 7; Supplemental Fig. S5). The clusters were more well-separated and equidistant for K-253 than K-676 (Supplemental Fig. S5). Although both performed well, the genotype-to-phenotype correlation was stronger for K-253 (${\hat{r}}_{\bar{y},M}=\mathrm{0.83}$) than K-676 (${\hat{r}}_{\bar{y},M}=\mathrm{0.67}$). This was consistent with the AX-184210676 SNP explaining less of the genetic variation and having a lower predictive value among the 43 long-read sequenced individuals than the AX-19424253 SNP (Table 1).

Of the three KASP assays designed for SNPs in the 5′-UTR of *PG1-6A1*, one was highly accurate (K-732), one failed, and the other was slightly less accurate and did not perform as well as K-732. K-732 genotypes were strongly correlated with fruit firmness phenotypes (${\hat{r}}_{\bar{y},M}=\mathrm{0.82}$). The genotype call rate for the K-732 assay (82.8%) was similar to that for K-SPM assay (86.0%), both of which were markedly lower than the genotype call rates observed for the K-676 and K-253 assays (Table 3; Fig. 7; Supplemental Fig. S5).

The KASP assay designed for the *En/Spm* INDEL (K-SPM) was less accurate than the K-732 and K-253 assays (Table 3; Fig. 7; Supplemental Fig. S5). The lower performance of that assay was presumably caused by the technical difficulty of amplifying DNA sequences bordering the 4948-bp insertion (Supplemental Fig. S4). Using long-read DNA sequences as a reference, genotyping errors for the K-SPM assay appear to have been primarily caused by mistyping insertion homozygotes as heterozygotes, which caused significant segregation distortion among the full-sib progeny phenotyped and genotyped in our study (Fig. 7; Supplemental Table S10).

The observed segregation ratios for the K-676, K732, and K-253 assays were not significantly different from the expected segregation ratio among the full-sib progeny genotyped in our KASP marker validation study (1 +/+: 2 +/−: 1 −/−): K-676 (χ^2^ = 2.88; *p* = 0.24), K-732 (χ^2^ = 0.44; *p* = 0.81), and K-253 (χ^2^ = 0.25; *p* = 0.88). The observed segregation ratio for the K-SPM assay (69 +/+: 51 +/−: 32 −/−), by comparison, was significantly distorted with an excess of favorable allele homozygotes and shortage of heterozygotes (χ^2^ = 34.36; *p* ≤ 0.0001). Although the *En/Spm* was homozygous and appears to be predictive of the favorable *PG1-6A1* allele found in modern, firm-fruited UC cultivars, the *En/Spm* INDEL proved to be more difficult to accurately genotype by KASP than the SNPs we targeted.

### The favorable *PG1-6A1* allele appears to be nearly fixed in a population with a long history of selection for increased fruit firmness

Using GBS- and KASP-called genetic variants, we discovered that the favorable *PG1-6A1* allele was nearly fixed in modern, firm-fruited UC cultivars and associated genetic resources, hereafter identified as the California population (Figs. 8–9). We discovered this in part by screening the parents of 178 full-sib families with three KASP markers: K-676, K-SPM, and K-253 (Supplemental Table S10). Using those KASP markers, the frequency of the favorable *PG1-6A1* allele was estimated to range from 0.93 to 0.97 among 10 650 full-sib individuals sampled from the California population in the 2023–24 cycle of selection. Of the 178 full-sib families, only four (23B660, 23B661, 23B760, and 23B762) originated from crosses where both parents were heterozygous for KASP marker alleles and predicted to segregate 1 *PG1-6A1*^+^/*PG1-6A1*^+^: 2 *PG1-6A1*^+^/*PG1-6A1*^−^: 1 *PG1-6A1*^−^/*PG1-6A1*^−^. Importantly, those were the only families in our survey of California population progeny in 2023–24 where the additive and dominance effects of the *PG1-6A1* locus could be estimated (where both parents were heterozygous for *PG1-6A1* alleles). These findings further substantiate that phenotypic selection alone has strongly swept the favorable *PG1-6A1* allele within the California population [5, 58].

Figure 7 displays the phenotypes of individuals observed within KASP marker genotypic classes in the diversity and full-sib populations. Our additive effect estimates for the *PG1-6A1* locus were nearly identical assay-to-assay and highly significant in the diversity population (0.09–0.10 kg-force), whereas the dominance effects were smaller, mostly nonsignificant, and slightly more variable (0.00–0.04 kg-force; Table 3; Fig. 7). The same pattern was observed in the full-sib population; however, the additive effect estimates were 50% smaller, and the KASP markers were 30–49% less predictive. Wildtype *PG1-6A1* homozygotes were firmer, and the dominance of the wildtype *PG1-6A1* allele was greater in the full-sib than the diversity population (Table 3; Fig. 7). Our degree-of-dominance ($\mid \hat{d}/\hat{a}\mid$) estimates for KASP markers associated with the *PG1-6A1* locus ranged from 0.02 (nearly additive) to 0.44 in the diversity population and 0.33 to 0.74 in the full-sib population, and increased as the dominance of the wildtype allele increased.

The assay-to-assay variation observed within populations was attributed to differences in KASP assay accuracy, identity-by-state variation, and stochastic variation (Table 3; Fig. 7). We attributed the dampened effect of the *PG1-6A1* locus in the full-sib population to genetic background effects (Table 3; Fig. 7). Although our analysis was limited to four full-sib families because of the high frequency of the favorable *PG1-6A1* allele, phenotypes observed within *PG1-6A1* genotypic classes suggest that favorable alleles for multiple QTL have been targeted by phenotypic selection and accumulated in the California population (Figs 5 and 7; Table 1 and 3). That conclusion is consistent with the finding that long-term selection appears to have virtually eliminated genetic variation for fruit firmness within the California population [5, 58], which we have shown was substantially caused by the fixation of the favorable *PG1-6A1* allele (Figs. 8–9).

### *PG1-6A1* loss-of-function mutations more than double fruit firmness in strawberry

The ecotypes and cultivars screened in our study spanned the domestication spectrum from extremely soft-fruited wild relatives to extremely firm-fruited cultivars (Table 3; Figs 7–9). Supplemental Fig. S6 displays the phenotypic means for 381 additional cultivars and other hybrid individuals not shown in Fig. 8. The firmest individual in our study (17C140P012), a favorable allele homozygote (*PG1-6A1*^+^/*PG1-6A1*^+^), withstood 0.45 kg-force of pressure before a 3 mm penetrometer probe broke through the surface of the receptacle (Fig. 8). The UC cultivars ‘Royal Royce’ (0.41–0.45 kg-force) and ‘Surfline’ (0.41–0.44 kg-force) were similarly firm. At the other extreme were three *F. virginiana* ecotypes with extremely soft, fragile, and easily bruised fruit (0.07 to 0.08 kg-force). They were predicted to be homozygous for wildtype (unfavorable) alleles (Supplemental Table S1 ).

The phenotypes of several *Fragaria chiloensis* and *F. virginiana* ecotypes are shown to the left of *F.*  $\times$  *ananassa* cultivars in Fig. 8 and Supplemental Fig. S6. Our analysis shows that ecotypes of the wild relatives are extremely to moderately soft-fruited (0.07 to 0.20 kg-force; $\tilde{y}=\mathrm{0.15}$). The fruit firmness medians ($\tilde{y}$) and maximums (${\bar{y}}_{MAX}$) were lower for *F. virginiana* ecotypes ($\tilde{y}=\mathrm{0.15}$ and ${\bar{y}}_{MAX}=\mathrm{0.18}$ kg-force) and *F. chiloensis* ecotypes ($\tilde{y}=\mathrm{0.16}$ and ${\bar{y}}_{MAX}=\mathrm{0.20}$ kg-force) than *F.*  $\times$  *ananassa* cultivars ($\tilde{y}=\mathrm{0.24}$ and ${\bar{y}}_{MAX}=\mathrm{0.45}$ kg-force) (Table 3; Figs 7 and 8; Supplemental Table S1 ). Statistics for the latter were estimated from the phenotypes of cultivars spanning the fruit firmness range, from ‘Sitka’ (PI616777; $\bar{y}=\mathrm{0.09}$ kg-force) and ‘Jucunda’ (PI551623; $\bar{y}=\mathrm{0.10}$ kg-force) at the lower extreme to ‘UC Surfline’ ($\bar{y}=\mathrm{0.41}$ kg-force) and ‘UC Royal Royce’ ($\bar{y}=\mathrm{0.44}$ kg-force) at the upper extreme. The fruit firmness median for modern UC cultivars (1988-present; $\tilde{y}=\mathrm{0.35}$ kg-force) was double that of heirloom cultivars ($\tilde{y}=\mathrm{0.18}$ kg-force) and slightly more than double that of wild species ecotypes (Fig. 8; Supplemental Table S1 ).

We suspect that the phenotypic minimums for *F. chiloensis* and *F. virginiana* ecotypes were overestimated (biased upward) because their fruit were often too soft to phenotype or even disintegrated when fully ripe. Nevertheless, using the phenotypic extremes observed in the diversity population, we estimated that domestication has increased fruit firmness by 628% from the softest wild species ecotypes (0.07 kg-force) to the firmest cultivars (0.45 kg-force). That genetic gain was substantially, but not solely driven by *PG1-6A1* mutations, which appear to double fruit firmness in most genetic backgrounds, e.g., the mean fruit firmness predicted by the KASP assay for the *En*/*Spm* INDEL was ${\bar{y}}_{+/+}=\mathrm{0.35}$ kg-force for the favorable allele homozygote and ${\bar{y}}_{-/-}=\mathrm{0.14}$ kg-force for the unfavorable allele homozygote (Table 3; Fig. 7; Supplemental Table S1 ).

The *PG1-6A1* genotypes predicted by the AX-184242253 SNP are depicted in a visual reconstruction of the history of breeding for increased fruit firmness in strawberry (Fig. 8–9). The phenotypic means ($\bar{y}$) displayed in Fig. 8 were estimated from a meta-analysis of phenotypic observations collected from field experiments over the course of our studies (Supplemental Fig. S6; Supplemental Table S1 ). The hockey stick (exponential growth) curve for fruit firmness uncovered by our analysis traces the increase in the frequency of the favorable *PG1-6A1* allele and favorable allele homozygotes (*PG1-6A1*^+^/*PG1-6A1*^+^) from the early 1950s onwards when moderately firm-fruited cultivars (*PG1-6A1* heterozygotes) began emerging and became catalysts for the expansion of strawberry production in California [5]. The change in fruit firmness was negligible from 1850 to 1950, apart from notable outliers, e.g., heirloom cultivars ‘Aberdeen’ and ‘Titan’ (Fig. 8).

### Outliers and breakdowns in the prediction accuracy of identical-by-state genetic variants

The SNPs targeted for marker development were highly predictive proxies for *PG1-6A1* alleles in modern and heirloom cultivars, but not 100% predictive across diverse germplasm (Table 1 and 3; Supplemental Table S9). Using five array-genotyped SNPs (AX-184953741, AX-184726882, AX-184210676, AX-184275052, and AX-184242253) as proxies for *PG1-6A1* genetic variants, the frequency of the wildtype *PG1-6A1* allele was estimated to range from 0.61 for the AX-184275052 SNP to 0.94 for the AX-184242253 SNP and 0.97 for the AX-184953741 SNP among 18 ecotypes of the wild relatives screened in our study (Supplemental Table S1 ). Using the AX-18424253 SNP, the *F. chiloensis* ecotype ‘ILE 02A’ (PI552038; 0.13 kg-force) and *F. virginiana* ecotype PI612320 (0.17 kg-force) were predicted to be heterozygous for *PG1-6A1* alleles. Their soft phenotypes, however, suggest that they are either homozygous for a wildtype alleles. Importantly, the *PG1-6A1* gene was more strongly expressed in ripe fruit of ‘ILE 02A’ than any of the *F.*  $\times$  *ananassa* cultivars screened by RT-PCR in our study (Fig. 4). These examples illustrate the breakdown in prediction accuracy of IBS genetic variants in strawberry, a highly polymorphic and heterozygous species. Although only five of the 18 array-genotyped ecotypes were long-read sequenced, they were found to be homozygous for the *En/Spm* deletion. The *En/Spm* INDEL appears to be highly predictive of wildtype and mutant *PG1-6A1* alleles that are nevertheless riddled with IBS genetic variants.

We suspect that the phenotype observed for ‘Aberdeen’ in our study (1910; PI551630; $\bar{y}=\mathrm{0.35}$ kg-force) could be erroneous (Supplemental Table S1 ). ‘Aberdeen’ was predicted to be homozygous for a wildtype *PG1-6A1* allele (Fig. 8; Supplemental Table S1 ), was soft-fruited (0.10 kg-force) in the [32] study, and was described by Darrow [2] as soft-fruited and “too soft to ship” (Supplemental Table S1 ). ‘Aberdeen’ could conceivably carry a favorable *PG1-6A1* allele different from the one found in modern UC cultivars or be a source of favorable alleles for loci other than *PG1-6A1*, but that seems unlikely.

‘Titan’ (PI551398; $\bar{y}=\mathrm{0.33}$ kg-force), a firm-fruited heirloom cultivar predicted to be heterozygous for *PG1-6A1* alleles, was as firm as many of the firm-fruited UC cultivars known to be homozygous for the favorable allele (Fig. 8). This cultivar could be heterozygous for SNPs in LD with different favorable *PG1-6A1* alleles or could carry favorable alleles at loci other than *PG1-6A1*. Although we systematically sampled genetic diversity in the UC and USDA genetic resource collections (https://www.ars-grin.gov/), deeper sampling in other collections might identify additional outliers and sources of novel favorable alleles and shed additional light on the domestication history of strawberry (Figs. 8–9).

### *PG1-6A1*, a single gene of pivotal importance to strawberry domestication

Our analyses show that the frequency of the mutant *PG1-6A1* allele gradually increased over two decades (1953–1973) in the UC breeding program before mutant homozygotes and LSL cultivars began emerging (Figs. 8–9; Supplemental Fig. S6; [5]). Their emergence played a critical role in the California strawberry Green Revolution and global expansion of strawberry production [58]. The phenotypic range for early cultivars was found to be virtually identical to that for wild species ecotypes in our study, most of which were predicted to be homozygous for the wildtype *PG1-6A1* allele (blue points in Fig. 8 and Supplemental Fig. S6; Supplemental Table S1 ). ‘Lassen’ (1935; 36C003P001; not phenotyped), a descendant of ‘Blakemore’ (1929; PI551421; $\bar{y}=\mathrm{0.18}$ kg-force), was the oldest UC cultivar predicted to be heterozygous for wildtype and mutant *PG1-6A1* alleles (Fig. 9; Supplemental Table S1 ). GBS- and KASP-called genotypes suggest that the favorable *PG1-6A1* allele was present but infrequent in early cultivars, was present in UC genetics from inception, and was inherited from ‘Blakemore’ (Fig. 9). We found that selection for increased fruit firmness in the early 1950s began exposing the effect of the incompletely dominant favorable *PG1-6A1* allele transmitted by ‘Tioga’ (1953; 53C009P002; $\overline{y}=\mathrm{0.25}$ kg-force) and ‘Tufts’ (1963; 63C120P011; $\bar{y}=\mathrm{0.25}$ kg-force), both of which are descendants of ‘Lassen’ and are the second oldest UC cultivars predicted to be heterozygous for *PG1-6A1* alleles (Figs. 8–9).

These early heterozygotes were superseded in the 1970s by the emergence of super firm-fruited *PG1-6A1*^+^ homozygotes with greatly increased shelf-life and decreased perishability, starting with the cultivars ‘Douglas’ (1972; 72C266P604; $\bar{y}=\mathrm{0.13}$ kg-force) and ‘Selva’ (1975; 75C071P107; $\bar{y}=\mathrm{0.30}$ kg-force). Digging deeper into the breeding history, we discovered that the favorable *PG1-6A1* allele found in modern UC cultivars was targeted by phenotypic selection in segregating populations as early as 1953 and increased in frequency until favorable *PG1-6A1* allele homozygotes began emerging approximately two decades later (Figs. 8–9). There are thousands of descendants of ‘Tioga’, ‘Tufts’, ‘Douglas’, and ‘Selva’ in UC pedigree records, which include every UC cultivar developed since 1970, all of which appear to be homozygous for the favorable *PG1-6A1* allele (Fig. 8–9; Supplemental Table S1 ). The abbreviated family tree developed for UC cultivars, from ‘Lassen’ and ‘Tioga’ to ‘Golden Gate’ and ‘Surfline’, illustrates the transition from soft- to firm-fruited phenotypes, the emergence and flow of the favorable *PG1-6A1* allele in UC parents and progeny, and the critical importance of the favorable *PG1-6A1* allele to strawberry domestication (Fig. 9).

## Discussion

We discovered that the fruit firmness QTL identified on chromosome 6A in previous genome-wide association and genetic mapping studies [21–24, 32, 58] was caused by a loss-of-function mutation in *PG1-6A1*, one of three tandemly duplicated paralogs of *FaPG1* [18], [25] (Figs. 1–4). The central importance of this gene in strawberry fruit softening was well established by findings in earlier transcriptomic and reverse genetics studies [18, 25–27]; however, until the octoploid genome was sequenced [31], genome-wide association studies could not be undertaken, the association between *PG1-6A1* and the chromosome 6A QTL was unknown, and the three *PG1* paralogs in LD with the QTL had not been discovered and physically mapped [32] (Fig. 2). Our study documented the central importance of *PG1-6A1* mutations to strawberry domestication and the expansion of production in California (Figs. 8–9), as predicted by earlier studies of *FaPG1* gene expression in developing fruit [18, 68].

Using an *F. virginiana* ecotype (PI552277, $\bar{y}=\mathrm{0.07}$ kg-force) and a UC hybrid (2017; 17C140P012; $\bar{y}=\mathrm{0.45}$ to 0.49 kg-force) from opposite tails of the phenotypic distribution, we estimated that breeding has increased fruit firmness by 643-700% in cultivated strawberry (Fig. 8; Supplemental Table S1 ). That estimate is remarkably close to the estimate of 768% reported for fruit firmness in a study of historical genetic gains from breeding in the California population [5]. Those genetic gains were driven by phenotypic selection for increased fruit firmness, which naturally drove the frequency of the favorable *PG1-6A1* upward (Fig. 8). The *PG1-6A1* mutation has been a critical component of those genetic gains, but clearly not the only component (Tables 1 and 3).

Compared to typical plant domestication time scales [69, 70], strawberry domestication has been anything but typical [2, 5, 32]. The most dramatic domestication changes in strawberry have arisen in the last half century [5, 32] and included the exponential change in fruit firmness caused by the fixation of the mutant *PG1-6A1* allele (Figs. 8 and 9). The speed with which mutant homozygotes emerged in the twentieth century seems slow by modern, genome-informed standards (Fig. 8), e.g., MAS and CRISPR/Cas9 editing of the *PG1-6A1* gene could conceivably collapse the process into a mere two years [25, 71, 72].

The expression of *PG1-6A1* in ripe fruit was found to be an accurate, albeit dominant biomarker for wildtype and mutant *PG1-6A1* alleles (Fig. 4). The differences observed in the expression of *PG1-6A1* using the qRT-PCR assay were insufficient to distinguish wildtype homozygotes from heterozygotes but unequivocally distinguished mutant homozygotes from wildtype homozygotes and heterozygotes (Fig. 4; Supplemental Table S9). Despite their predictive ability, qRT-PCR assays require the isolation of RNA from fruit-bearing plants and are impractical for MAS, especially for a trait like fruit firmness that can be rapidly and inexpensively phenotyped once plants begin bearing fruit. The SNPs and *En/Spm* INDEL that we selected for marker development were strong but imperfect predictors of *PG1-6A1* genotypes because of identity-by-state and nongenetic variation, including genotyping errors (Tables 1, 2, and 3; Supplemental Table S1 ). Our search for codominant DNA-based biomarkers as accurate and predictive as dominant RNA-based biomarkers was motivated by the prospect of applying MAS on a large-scale to DNA isolated from seedlings early in plant development, thereby circumventing the need to grow plants to physiological maturity to firmness, increasing breeding throughput, and decreasing greenhouse and field footprints (Table 3).

Although the favorable *PG1-6A1* allele had been driven to near fixation in UC LSL breeding populations, thereby diminishing the need for MAS, wildtype *PG1-6A1* alleles have been reintroduced into LSL genetic backgrounds over the last 10 years by wild species and heirloom cultivar introgressions, as exemplified by the full-sib families screened in our study (Fig. 7). We suspect that novel favorable alleles for fruit firmness have been introduced by the exotic donors (parents), e.g., the firmness of 20B239P024 (0.28 kg-force), a putative *PG1-6A1* heterozygote, was similar to that of ‘Petaluma’ and ‘Grenada’, UC cultivars at the lower end of the fruit firmness range among favorable allele homozygotes (Fig. 8). That hybrid (20B239P024) has a moderately firm-fruited grandparent (‘Puget Reliance’; 0.14 kg-force) and firm-fruited UC cultivars as the other grandparents.

We hypothesize that the *PG1-6A1* loss-of-function mutation found in modern, firm-fruited UC cultivars was caused by the insertion of the *En*/*Spm* TE upstream of *PG1-6A1* (Supplemental Fig. S4). *En*/*Spm* TEs are important mediators of phenotypic diversity in plants [52, 53, 56, 57, 73], and often create novel phenotypes by disrupting the function or modifying the expression of genes through insertional mutagenesis in coding or promoter sequences. Although numerous TE insertional mutations have been described in plants, and their effects are often predictable, the mutant phenotype observed in our study was not caused by an insertional mutation in *PG1-6A1 per se* [41, 56, 73]. Castillejo et al. [74] discovered an *En*/*Spm*-2 TE insertion in the promoter of *MYB10–2*, an R2R2 MYB transcription factor that regulates anthocyanin biosynthesis in strawberry. They showed that the mutation (TE insertion) enhanced the expression of *MYB10–2* and increased the accumulation of anthocyanins in ripe fruit. This appears to be the only TE insertional mutant reported to date in strawberry. Apart from insertional mutagenesis, TEs create novel phenotypes by altering the regulation of nearby genes (reviewed by [41]). A TE inserted upstream of a nearby gene can “insert promoter sequences and introduce an alternative transcription start site, disrupt an existing cis-regulatory element, or introduce a new cis element such as a transcription factor binding site” [41]. We suspect that one of these mechanisms underlies the *PG1-6A1* expression variation and mutant phenotypes observed in populations segregating for the *En*/*Spm* TE insertion (Supplemental Fig. S4).

The increases in fruit firmness associated with anti-sense silencing of *PG1* and gene-edited knockdown mutations of *PG1* in the cultivar ‘Chandler’ [25–27] appear to be slightly less substantial than those reported for naturally occurring *PG1* loss-of-function mutations in our studies (Figs 5, 7, and 8; Tables 1, 2, and 3; Supplemental Table S1 ). We observed a 206–265% increase in fruit firmness between the soft-fruited cultivar ‘Chandler’ ($\bar{y}=\mathrm{0.17}$ kg-force; *PG1-6A1*^−/^*PG1-6A1*^−^) and the firm-fruited cultivar ‘Royal Royce’ ($\bar{y}=\mathrm{0.35}$ to 0.45 kg-force; *PG1-6A1*^+^/*PG1-6A1*^+^). The fruit firmness increases reported by Paniagua et al. [26] from anti-sense silencing of *PG1* ranged from 0 to 140% (approximated by us from phenotypic means of *PG1* transgenic plants displayed in their bar chart). Similarly, Lopez-Casado et al. [25] reported increases in fruit firmness in the 37–70% range among transgenic plants carrying knockdown edited *PG1* genes. These forward and reverse genetic studies clearly substantiate the importance of the *PG1-6A1* QTL to strawberry domestication, and the development of LSL cultivars in particular.

Our analyses show that the favorable *PG1-6A1* allele is necessary and often sufficient to achieve the firmness needed for production in markets where the fruit is cold-stored and shipped medium to long distances. Nevertheless, several findings in the present and previous studies clearly show that multiple QTL must underlie the heritable variation observed for fruit firmness in strawberry. First, the phenotypic range was wide among individuals predicted to be homozygous for the favorable *PG1-6A1* allele, which strongly suggests that multiple fruit firmness QTL could be segregating in the genetic backgrounds we studied (Figs 5 and 7). Second, the effect of the *PG1-6A1* locus was shown to be genetic background dependent (Figs 5 and 7; Tables 1 and 3), another strong indicator of the importance of additional QTL. Third, multiple fruit firmness QTL have been identified in previous GWAS and genetic mapping studies [21–24, 58]. While some of these could be false positives, future studies are bound to identify and validate additional QTL with predictable effects across environments and genetic backgrounds.

Fourth, our QTT analysis identified several genes that were differentially expressed between soft- and firm-fruited individuals and are either known or predicted to affect fruit development and softening (Fig. 2; Supplemental Table S4). That analysis identified compelling candidate genes for further study (Supplemental Table S4); however, genotype-to-phenotype associations were not observed for any of them in our study (Fig. 2), the previous study of Hardigan et al. [32], or our reanalysis of the latter using additional phenotypic observations (*n* = 5104; see Supplemental Fig. S1). Moreover, we could not identify any associations between QTT-discovered candidate genes (Supplemental Table S4) and previously identified QTL [21–24, 58].

Fifth, *POLYGALACTURONASE2* (*PG2*) was not among the genes identified in our forward genetic studies, but could be one of the genetic factors underlying the missing heritability for fruit firmness [26]. Paniagua et al. [26] showed that fruit firmness could be increased by transgenic anti-sense silencing of *PG2* in combination with *PG1*, and that *PG2* was more weakly expressed than *PG1* in ripe fruit, but expressed nonetheless. Consistent with our findings, Sanchez-Sevilla et al. [75] did not observe the expression of *PG2* in ripe fruit of the firm-fruited cultivar ‘Camarosa’ (shown to be homozygous for the *PG1-6A1*^+^ allele in the present study), whereas *PG1* was strongly expressed.

Lastly, the phenotypic ranges within *PG1-6A1* marker genotypic classes were wide and overlapping, e.g., fruit firmness ranged from 0.28 to 0.45 kg-force among individuals predicted to be homozygous for the mutant *PG1-6A1* allele using GBS and KASP markers (Figs 5, 7, and 8; Tables 1 and 3). That pattern was consistent with the finding that a fraction of the heritability for fruit firmness observed in our study was caused by the segregation of QTL that have not yet been identified or validated or have effects that are too small to be detected [21–24, 58]. Their identification could further enhance the prediction of fruit firmness phenotypes in strawberry beyond that predicted by genetic variants associated with *PG1-6A1*.

## Materials and methods

### Plant material and phenotyping

The fruit firmness (kg-force) phenotypes reported in our paper were recorded from samples of ripe fruit harvested from 178 individuals grown in field experiments in Oxnard, Santa Maria, Davis, and Winters, California, and a greenhouse experiment in Davis, California. The number and composition of individuals differed across these experiments (documented in Supplemental Table S1 ). They included 69 *F.*  $\times$  *ananassa* cultivars developed between 1854 and 2017, 91 clonally propagated hybrid individuals with diverse *F.*  $\times$  *ananassa* parentage developed at UC Davis, four *F. chiloensis* ecotypes, 13 *F. virginiana* ecotypes, and one *F. vesca* ecotype. Of the 91 hybrid individuals, 12 were developed between 1953 and 2012, and 79 were developed between 2016 and 2020. Of the latter, 43 originated from crosses between elite, firm-fruited UC parents and soft-fruited non-UC heirloom cultivars, whereas 36 originated from crosses between elite, firm-fruited UC parents (Supplemental Table S1 ).

The firmness of ripe fruit (kg-force) was measured using a TA.XT Plus Texture Analyzer fitted with a TA-53 3-mm puncture probe (Stable Micro Systems Ltd., Goldalming, United Kingdom). The fruit were positioned horizontally on the instrument platform such that the probe targeted receptacle tissue near the latitudinal center.

The firmness of ripe fruit (kg-force) was measured using a FR-5120 Digital Fruit Firmness Tester (penetrometer) fitted with a 3 mm puncture probe (Stable Micro Systems Ltd., Goldalming, United Kingdom). The penetrometer was held in a stand with the probe point upward, and fruit were lowered horizontally onto the instrument such that the probe targeted receptacle tissue near the latitudinal center.

### Discovery population

The phenotypes for our GWAS, QTT, and expression-QTL analyses were collected from 85 *F.*  $\times$  *ananassa* individuals grown in Santa Maria and Oxnard, California field experiments in 2020–21 and 2021–22 (hereafter the ‘discovery’ population). They included 13 cultivars and 72 other hybrid individuals from the UC strawberry breeding program (identified in Supplemental Table S1 ). They were arranged in randomized complete blocks experiment designs and grown on commercial farms using standard management practices. The experimental units were 20-plant plots with plants equally spaced in raised beds planted with diagonal staggering of plants in three rows to a density of 54 362 plants/hectare. The bare-root plants (clones) for these experiments were produced in a commercial nursery (Cedar Point, Dorris, CA) between April and October of each planting year. They were harvested and placed in cold storage (4°C) for less than one week before being planted at the Oxnard and Santa Maria field sites. Three ripe fruit were harvested from each plot in March and April of each year, placed in cold storage (4°C) for less than 24 h, and phenotyped as described above. Across-year phenotypic means were estimated from 24 observations/individual (2 replications/individual $\times$ 3 samples/replication $\times$ 2 harvests/year $\times$ 2 years).

The transcriptomes of the 85 discovery population individuals were analyzed using RNA samples isolated from ripe fruit harvested in March, 2021 from three 20-plant plot replications/individual in both locations. Those data were previously analyzed and described by Fan et al. [76] and are available in the NCBI Short-Read Archive under Bioproject #PRJNA787565.

### Diversity population

We assembled and phenotyped a genetically diverse collection of clonally propagated hybrid individuals ($n=\mathrm{92}$) for the identification of genetic variants associated with a fruit firmness QTL on chromosome 6A, the identification of genetic variants associated with gene expression differences in different stages of fruit development, the development and validation of high-throughput genotyping assays for SNPs and INDELs, specifically KASP assays [77], and analysis of the domestication history of LSL cultivars. These individuals were chosen to sample the widest possible range of fruit firmness phenotypes and for testing hypotheses in different studies.

We used data for every individual and different subsets of individuals for specific analyses. The subsets included eight individuals used for a quantitative RT-PCR study (see below), 43 individuals used for an analysis of genetic variants among long-read DNA sequences (see below), and 101 individuals for a meta-analysis of fruit firmness phenotypes across experiments. Those included phenotypes of 85 individuals collected for the discovery population (see above), phenotypes collected from individual field grown plants of 87 individuals preserved in the UC Davis Strawberry Germplasm Collection at the Wolfskill Experiment Orchard (WEO), Winters, California in 2020–21 and 2021–22, and phenotypes of three clones/individual of 43 long-read DNA sequenced individuals grown in a UC Davis greenhouse in 2023 (identified in Supplemental Table S1 ). We phenotyped three fruit/harvest on six harvest dates in April and May 2021 and four harvest dates in April and May 2022 at WEO and three fruit/clone on five harvest dates in the greenhouse experiment. The data collected for the 87 individuals at WEO were used for KASP marker testing and validation.

### Full-sib families

We selected four full-sib families from 178 crosses within the 2023–24 breeding pipeline that showed three genotypic classes using K-676, K-SPM, K-732, and K-253. We harvested all ripe fruits from one-year-old, seed-propagated plants grown at WEO ($n=\mathrm{152}$) across two harvest dates in May, with an average collection of two and four ripe fruits per individual, respectively. Fruit firmness was measured using the previously described protocol.

EMMs were calculated for one-year-old, seed-propagated plants through the *sommer* R package [78] and *emmeans* R packages [79]. The linear mixed model (LMM) was formulated as:


(1) \begin{equation*} {y}_{hg}={H}_h+{G}_g+{e}_{hg} \end{equation*}

where ${y}_{hg}$ represents the observed phenotype for the ${g}^{th}$ genotype in the ${h}^{th}$ harvest. ${G}_g$ is the effect of the ${g}^{th}$ genotype, and ${H}_h$ is the random effect of the ${h}^{th}$ harvest time.

### GWAS population

Fruit firmness (kg-force) was measured on 460 wild and domesticated individuals in 2018 from replicated 6-plant plots grown in Ventura, CA under commercial field conditions. The composition of the GWAS population is shown in Supplemental Table S1 . The data from the first harvest were first reported by Hardigan et al. [32]. Fruit firmness was measured on six randomly selected berries from each plot from two harvests in March and April of 2018 as the maximum force with a QA Supplies FT2 handheld penetrometer equipped with a 3 mm probe (QA Supplies, Norfolk, VA, USA) [80]. These individuals ($n=\mathrm{460}$) were genotyped with a 50 K SNP array [33].

EMMs were calculated for this population using the *lme4* [81] and *emmeans* R packages [79]. The LMM was formulated as:


(2) \begin{equation*} {y}_{hgbf}={H}_h+{B}_b+{G}_g+G{B}_{gb}+{e}_{hgbf} \end{equation*}

where ${y}_{hgbf}$ represents the ${f}^{th}$ fruit phenotype for the ${g}^{th}$ genotype in the ${b}^{th}$ block during the ${h}^{th}$ harvest. In this model, ${H}_h$ denotes the fixed effect of the ${h}^{th}$ harvest, ${G}_g$ is the fixed effect of the ${g}^{th}$ genotype. ${B}_b$ is the random effect of the ${b}^{th}$ block, $G{B}_{gb}$ represents the random effect of the interaction between the ${g}^{th}$ genotype and the ${b}^{th}$ block (i.e., plot and experimental unit), and ${e}_{hgbf}$ is the residual effect of $hgb{f}^{th}$ fruit (i.e. subsample).

### Statistical analyses

Initial analysis of the raw phenotypic data was conducted using a LMM through the *sommer* R package [78]. EMMs were calculated for the UCD breeding collection via the *emmeans* R package [79]. The LMM across years was formulated as:


(3) \begin{equation*} {y}_{lgyb}={L}_l+{G}_g+{Y}_y+L{Y}_{ly}+G{Y}_{gy}+ LY{B}_{ly b}+{e}_{lgyb} \end{equation*}

where ${y}_{ijkl}$ is the observed phenotype for the ${g}^{th}$ genotype in the ${l}^{th}$ location during the ${y}^{th}$ year at the ${b}^{th}$ complete block. ${L}_l$ indicates the effect of the ${l}^{th}$ location, ${G}_g$ is the effect of the ${g}^{th}$ genotype. ${Y}_y$ is the random effect of the ${y}^{th}$ year, $L{Y}_{ly}$ is the random effect of the interaction between the ${l}^{th}$ location and the ${y}^{th}$ year, $G{Y}_{gy}$ is the random effect of the ${g}^{th}$ genotype in the ${y}^{th}$ year, $LY{B}_{lyb}$ indicates the random effect of the interaction among the ${l}^{th}$ location, ${y}^{th}$ year, and ${b}^{th}$ complete block, and ${e}_{lgyb}$ is the residual effect.

Variance components were calculated for the random effects in LMM using the restricted maximum likelihood (REML) method. Broad-sense heritability on a clone-mean basis was estimated by ${\hat{H}}^2={\hat{\sigma}}_G^2/{\hat{\sigma}}_P^2$, where ${\hat{\sigma}}_G^2$ is the genetic variance among clonally propagated individuals (genotypes), ${\hat{\sigma}}_{\overline{P}}^2={\hat{\sigma}}_G^2+{\hat{\sigma}}_{GxY}^2/y+{\hat{\sigma}}_e^2/ ry$ is the phenotypic variance on a clone-mean basis, ${\hat{\sigma}}_{GxY}^2$ is the genotype $\times$ year interaction variance, ${\hat{\sigma}}_e^2$ is the residual variance, $y$ is the number of years, and $r=\mathrm{11.23}$ is the harmonic mean of the number of replications per genotype across years. Narrow-sense heritability was estimated by ${\hat{h}}^2={\hat{\sigma}}_A^2/{\hat{\sigma}}_P^2$, where ${\hat{\sigma}}_A^2$ is an estimate of the additive genetic variance from an RR-BLUP analysis [82, 83] and ${\hat{\sigma}}_{\overline{P}}^2$ is the phenotypic variance on a clone-mean basis.

### GWAS

A single locus analysis for the across-year EMMs was performed using GEMMA v0.98.1 [84, 85]. This analysis incorporated 49 330 Axiom array SNP markers mapped in the ‘Royal Royce’ reference genome. The genomic relationship matrix ($\mathbf{K}$), derived from SNP marker genotypes [86], was used to adjust for genetic relationships among individuals [84, 85]. Percentage of genetic variance explained (GVE = ${\hat{\sigma}}_M^2/{\hat{\sigma}}_G^2$) and phenotypic variance explained (PVE = ${\hat{\sigma}}_M^2/{\hat{\sigma}}_P^2$) by the most significant SNPs were calculated, where ${\hat{\sigma}}_M^2$ is the bias-adjusted REML semi-variance estimate for the SNP marker, and ${\hat{\sigma}}_G^2$ and ${\hat{\sigma}}_P^2$ represent respective clone-mean-based genetic and phenotypic variance [87].

### DNA sequence analyses

We analyzed the structure and function of the Fxa6Ag103973 gene model in the Royal Royce genome using the Integrative Genomics Viewer v2.16.2 [88] and the InterPro database (https://www.ebi.ac.uk/interpro/; [89]), respectively. We used BLASTN 2.12.0+ [90], implemented by the Genome Database for Rosaceae (http://www.rosaceae.org; [91]), to identify homologous sequences for the first and last four exons, including the large intron between them in the Fxa6Ag103973 gene model. The queried sequences were aligned against the reference genomes ‘Camarosa’ v1.0.a1 [31], ‘Camarosa’ v1.0.a2 [42] and *Fragaria vesca* v4.0.a1 [46]. Genome-wide homologs of the curated Fxa6Ag103973 gene were inferred and visualized with JCVI v1.3.6 [92].

### Phylogenomic analyses of PG genes

PG genes were identified in the ‘Royal Royce’ reference genome using the gene ontology terms for PG activity (GO:0004650). PG genes exhibiting high sequence similarity to those within the firmness locus on chromosome 6A were detected via BLAST v2.15 [93]. Local synteny analysis was performed on homoeologous *PG1* genes [92]. Alignments of amino acid sequences for *PG1* paralogs and homoeologs identified in this study were performed with Clustal Omega (https://www.ebi.ac.uk/Tools/msa/clustalo/; [94]). the Interactive Tree Of Life v6 (https://itol.embl.de) was used to visualize the evolutionary relationships among these PGs.

### QTT analysis

Transcriptomic data from the discovery collection was aligned against the Royal Royce reference genome through STAR v2.7.10a [95]. Normalization, filtration, and conversion to ${\log}_2$-scaled counts per million (${\log}_2$ CPM) were performed using calcNormFactors(), filterByExpr(), and cpm() functions from the *edgeR* package in R [96], respectively. We further adjusted the data by including the $\mathbf{K}$ matrix in the polygenic function from the *GenABEL* package in R [97]. A Pearson’s correlation analysis of gene expression profiles (60 685 transcripts) and EMMs of firmness values was conducted using the cor.test function in R. We applied a threshold of 0.005 to identify transcripts as QTTs when this stringent criterion was met.

### Gene expression analyses

Expression profiles of the curated Fxa6Ag103973 gene model were analyzed in the firm-fruited cultivar ‘Royal Royce’ and the soft-fruited cultivar ‘Mara des Bois’ across the unripe, white, and ripe fruit development stages. Total RNA was isolated from pooled three ripe fruits after flash-freezing in liquid nitrogen using the Quick-RNA Miniprep Kit from Zymo Research. Sequencing on the Illumina NovoSeq platform yielded an average of 17.6 Gb per sample. We aligned the sequencing reads to the updated gene annotation of the ‘Royal Royce’ reference genome. Subsequently, transcript abundances were normalized to ${\log}_2$ CPM and visualized with the pheatmap package in R [98].

### Real-time quantitative RT-PCR analyses

We quantified the expression levels of *PG1-6A1*, *PG1-6A2*, and *PG1-6D1* to evaluate their role in fruit firmness during ripening. Eight accessions were selected based on their firmness phenotypes and known genetic information concerning the *En/Spm* element upstream of the *PG1-6A1* gene (Supplemental Table S1 ). The accessions were grown at a UCD greenhouse facility in 2023 and represented by three biological replicates in a completely randomized design. We harvested three fruits for each biological replicate and pooled them for RNA isolation. After flash-freezing the samples in liquid nitrogen, the RNA extraction process was carried out using the Quick-RNA Miniprep Kit from Zymo Research with minor modifications. A precleaning procedure was considered from [99–101] before using the RNA extraction kit. Initially, samples were vortexed with 5 ml of a warmed extraction buffer containing 2-mercaptoethanol (100 μl) and incubated for 5 min at 65°C. The extraction buffer was made with 8 g cetyltrimethylammonium bromide 2% v/v, 8 g polyvinylpyrrolidone 2% v/v, 40 ml tris–HCL 100 mM, 160 ml NaCl 5 M, 20 ml ethylenediaminetetraacetic acid disodium salt 25 mM, and 0.2 g spermidine. We added deionized water to complete a final volume of 400 ml. 5 ml of chloroform–isoamyl alcohol (CIA 24:1) was added and mixed to centrifuge at 4000 rpm for 30 min at 4°C. The supernatant was transferred into a new tube, and an equal amount of CIA was added. The samples were vortexed and centrifuged again. The supernatant was transferred to a new tube, and 1/10 volume of potassium acetate 1 M was added and mixed well by inversion. Samples were centrifuged at 4000 rpm for 20 min at 4°C. The supernatant was transferred into a new tube, and 1/3 volume of lithium chloride was added and mixed well by inversion. Tubes were placed at −20°C overnight. The RNA extractision kit was conducted on day two. First-strand cDNA from total RNA extraction was synthesized through the RevertAid First Strand cDNA Synthesis Kit (Thermo Fisher Scientific, Inc.).

We established a DNA dilution of 1:125 for qPCR after analyzing the amplification efficiency for each primer set through a series of dilution assays (Supplemental Fig. S7). Details of the primers designed for each of the three PG genes with Primer3 v0.4.0 [102] are provided in Supplemental Table S9. We prepared the DNA template mixtures to a final volume of 10 $\mu$l, which included 4 $\mu$l DNA template, 1 $\mu$l of 10 $\mu$M primer mix, and 5 $\mu$l PowerUp^TM^ SYBRp^TM^ Green Master Mix (Applied Biosystemp^TM^). Amplifications were carried out on a QuanStudiop^TM^ 3 Real-Time PCR System (Applied Biosystemp^TM^) under the following thermal profile: initial denaturation at 95°C for 10 min, 40 amplification cycles at 95°C for 15 s and 60°C for 1 min, and followed by a dissociation curve analysis ramping from 60°C to 95°C at 1.6°C/s. We considered three technical replicates per sample and included water as a no-template control in every run. The mRNA levels were normalized to the expression of *Fragaria*  $\times$  *ananassa* DNA binding protein [103]. To elucidate the relationship between each PG gene and fruit firmness at different developmental stages, we calculated Pearson’s correlations in R.

### Long-read DNA sequencing and genetic variant analyses

We used highly accurate long sequencing reads (HiFi reads) from a diverse assemblage of 43 strawberry accessions (Supplemental Table S1 ), including wilds, heirlooms, advanced selections, and modern cultivars, to identify genomic variants in the *PG1* locus. HiFi reads were aligned to the ‘Royal Royce’ reference genome by using the pbmm2 tool [104]. We delimited the analysis of structural variations within the *PG1* locus via samtools view from Samtools v1.13 [105]. Subsequently, the identification and characterization of structural variation signatures were executed through the pbsv discover and pbsv call functions, available in pbsv v2.8.0 (https://github.com/PacificBiosciences/pbsv).

The INDELs found upstream of the *PG1-6A1* gene’s promoter were quantitatively associated with firmness metrics. The fruit firmness of the diverse strawberry panel grown at a UCD greenhouse facility was measured in 2023. We computed EMMs from five biological replicates and three ripe fruits per accession using a completely random experiment design across five harvest times (Supplemental Table S1 ). Pearson correlation coefficients and the corresponding *p*-values were calculated using R statistical software. In addition, we examined the identity of the INDEL located at −3926 bp from the ATG codon of the *PG1-6A1* gene using the CENSOR web server (https://www.girinst.org/; [106]).

We identified SNPs within the *PG1-6A1* gene using DeepVariant v1.4 [107]. SNPs were filtered using BCFTools v1.19 [105] with the view command, applying a minor allele frequency threshold of $\le$ 0.05 and a quality score threshold of $\ge$ 30. SNPs identified in the *PG1-6A1* gene were classified using SnpEff v5.2c [50]. We used SnpSift v5.2c [51] to identify SNPs significantly associated with fruit firmness by coding soft-fruited accessions as 1 (wildtype) and firm-fruited accessions as 2 (mutant). Additionally, cis-eQTLs were identified within the discovery population using the *MatrixEQTL* package in R [108], following the approach described by [76]. For further analysis, we focused on the most significant cis-eQTL associated with the *PG1-6A1* gene.

### DNA marker development

Four KASP assays were designed to target the *PG1* locus in a diverse strawberry panel (*n* = 92; Supplemental Table S1 ) and one-year-old, seed-propagated plants grown in 2023–24 ($n=\mathrm{152}$; Supplemental Table S10). The design of KASP markers was undertaken using the services provided by LGC Biosearchp^TM^ Technologies. We aimed to target the GWAS peak SNP, the most significant eQTL peak SNP, the *En/Spm-1* TE, and an intragenic SNP variant in the *PG1-6A1* coding sequence (Supplemental Table S9).

We established a statistical relationship between firmness EMMs and KASP markers by conducting Pearson’s correlation in the R platform. The additive and dominance effects were estimated by $\hat{a}=({\hat{\mu}}_{PG1-6A{1}^{-}/ PG1-6A{1}^{-}}-{\hat{\mu}}_{PG1-6A{1}^{+}/ PG1-6A{1}^{+}})/2$ and $\hat{d}={\hat{\mu}}_{PG1-6A{1}^{+}/ PG1-6A{1}^{-}}- \big({\hat{\mu}}_{PG1-6A{1}^{-}/ PG1-6A{1}^{-}}+{\hat{\mu}}_{PG1-6A{1}^{+}/ PG1-6A{1}^{+}}\mathrm{\big)/2}$, where ${\hat{\mu}}_{PG1-6A{1}^{-}/ PG1-6A{1}^{-}}$, ${\hat{\mu}}_{PG1-6A{1}^{+}/ PG1-6A{1}^{-}}$, and ${\hat{\mu}}_{PG1-6A{1}^{+}/ PG1-6A{1}^{+}}$ are the EMMs for individuals with $PG1-6A{1}^{-}/ PG1-6A{1}^{-}$, $PG1-6A{1}^{+}/ PG1-6A{1}^{-}$, and $PG1-6A{1}^{+}/ PG1-6A{1}^{+}$ genotypes for each KASP marker. $PG1-6A{1}^{-}$ is the unfavorable allele (wildtype), while $PG1-6A{1}^{+}$ is the favorable allele (mutant). The degree of dominance of each KASP marker was estimated by $\mid \hat{d}/\hat{a}\mid$ [109, 110]. We calculated the accuracy of KASP markers based on SNPs and the *En/Spm* element using data from the 50 K array (*n* = 90) and HiFi data (*n* = 37), respectively.

## Data Availability

The phenotypic and genotypic data and supplemental material for these studies are available in a DRYAD repository (10.5061/dryad.k3j9kd5hk). The long-read DNA sequences developed for this study have been deposited in the National Center for Biotechnology Information Sequence Read Archive under Bioproject #PRJNA1128720 (https://www.ncbi.nlm.nih.gov/bioproject/PRJNA1128720).
